# Emotional and Directional Enabled Programmable Flexible Haptic Interface for Enhanced Cognition in Disabled Community

**DOI:** 10.34133/research.0714

**Published:** 2025-06-03

**Authors:** Yuhan Liu, Liuyang Han, Siqi Lv, Tao Jiang, Mingkai Duan, Hanyu Guo, Yuzhen Li, Qisen Xie, Yanru Chen, Dongkai Wang, Ziheng Liu, Wenjie Zhang, Yanting Gong, Junwen Zhong, Xiang Qian

**Affiliations:** ^1^ Tsinghua Shenzhen International Graduate School, Tsinghua University, Shenzhen 518055, China.; ^2^ Department of Electromechanical Engineering, Centre for Artificial Intelligence and Robotics, University of Macau, Macau 999078, China.

## Abstract

Advanced haptic feedback interfaces are essential for human–machine interaction, particularly in assistive technologies that offer versatile commands, simplify navigation, and enhance emotional interactions for individuals with visual and hearing impairments. Current systems, primarily reliant on Braille and mechanical announcements, fall short of addressing these needs. Existing haptic interfaces, while providing various haptic feedback modes, still face challenges including rigid, strong current, or high-voltage stimulation, limiting their applicability and long-term safety for widespread use. Here, the work demonstrates a flexible, integrable, and programmable haptic interface based on elastomer actuators that utilize a unique forming process to create customized local stiffness in a multilayer elastomer by varying the cross-linking density of elastomers. Complemented by tailored software, the system delivers a 4-dimensional haptic experience within a safe voltage of less than 50 V and a frequency range of 50 to 450 Hz, enabling high-fidelity emotional and navigational feedback. Demonstrations of this system achieve an average accuracy rate of 64.6% in emotional interactions without prior training, improving to 95.8% with learning mode, along with an average accuracy rate of 94.2% for 9 directional commands in navigation interactions.

## Introduction

The development of haptic technology prompts the exploration of interactions between humans and the digital world, resulting in new applications in virtual reality [1–3], augmented reality [4–8], disability assistance [9,10], and communication [11]. Compared with state-of-the-art haptic sensors [12–14], advanced haptic actuators actively provide programmable touch sensations, delivering diverse feedback information to visually impaired or hearing-impaired individuals. This human-centered approach reduces reliance on a single sense, helps them prevent sensory imbalances, and mitigates outdoor hazards and safety concerns associated with sensory occupation. Haptic feedback technology is also expected to assist in social interaction and emotional communication, both of which are vital to human activities. Hearing-impaired individuals often struggle with traditional verbal communication, making it challenging to convey or understand emotions effectively through text. While visually impaired individuals can access information through devices that convert text to sound or Braille, they may still miss the emotional nuances present in face-to-face interactions.

Surface haptic technology is currently implemented through 2 main mechanisms: electrical stimulation and mechanical vibration. Electrical stimulation directly utilizes the electrical currents to stimulate the human nerves, which then transmit haptic signals to the brain [15]. However, this approach presents challenges [16,17] such as inconsistency in user experience, skin pain, muscle reactions, and temporary desensitization. In contrast, flexible mechanical actuators provide a safer, noninvasive alternative that conveys haptic information through the deformation of the skin surface [18]. Unlike nerve stimulation, this method does not require a firm attachment between the user’s hand and the haptic interface, allowing for greater freedom in user–environment interactions, and it eliminates the need for adjustments to ensure consistency across different users. However, flexible mechanical actuators typically utilize soft functional materials such as dielectric elastomers [19,20], piezoelectric materials [21,22], stimulus responsive materials [23–25], and electrets [11,26]. These actuators often depend on high-voltage power supplies in the kilovolt range, which pose substantial safety hazards and hinder the development of wireless haptic interfaces and multidimensional programming capabilities. Therefore, there is a growing demand for haptic interfaces that integrate low-voltage-driven actuators with high programmability to convey complex emotions and directional feedback.

In this paper, we present a highly programmable and low-voltage-driven haptic interface based on the multilayer variable-stiffness polydimethylsiloxane (PDMS) elastomer actuators. These elastomeric polymers, commonly used in flexible electronics, offer excellent stretchability [27,28] and transparency [29] but often exhibit high damping [30–32] and unchangeable stiffness, limiting their effectiveness in electro-mechanical applications. To address these limitations, this work introduces PDMS elastomers with variable stiffness, achieved by varying the amount of the ethoxysilanes used as the cross-linking agent to promote cross-linking reactions between molecular chains, thus tailoring its mechanical properties. The concept of variable-stiffness elastomers, achieved by selectively varying the cross-linking density of elastomers, was previously adopted and validated by Wang et al. [33] for developing intrinsically stretchable transistors and circuits. Here, we adopt a similar strategy for constructing a unique low-damping and enhanced elasticity soft material, in which the PDMS elastomer serves as a stiffness regulator, reducing the driving voltage from thousands of volts to below 50 V.

**Fig. 1.**
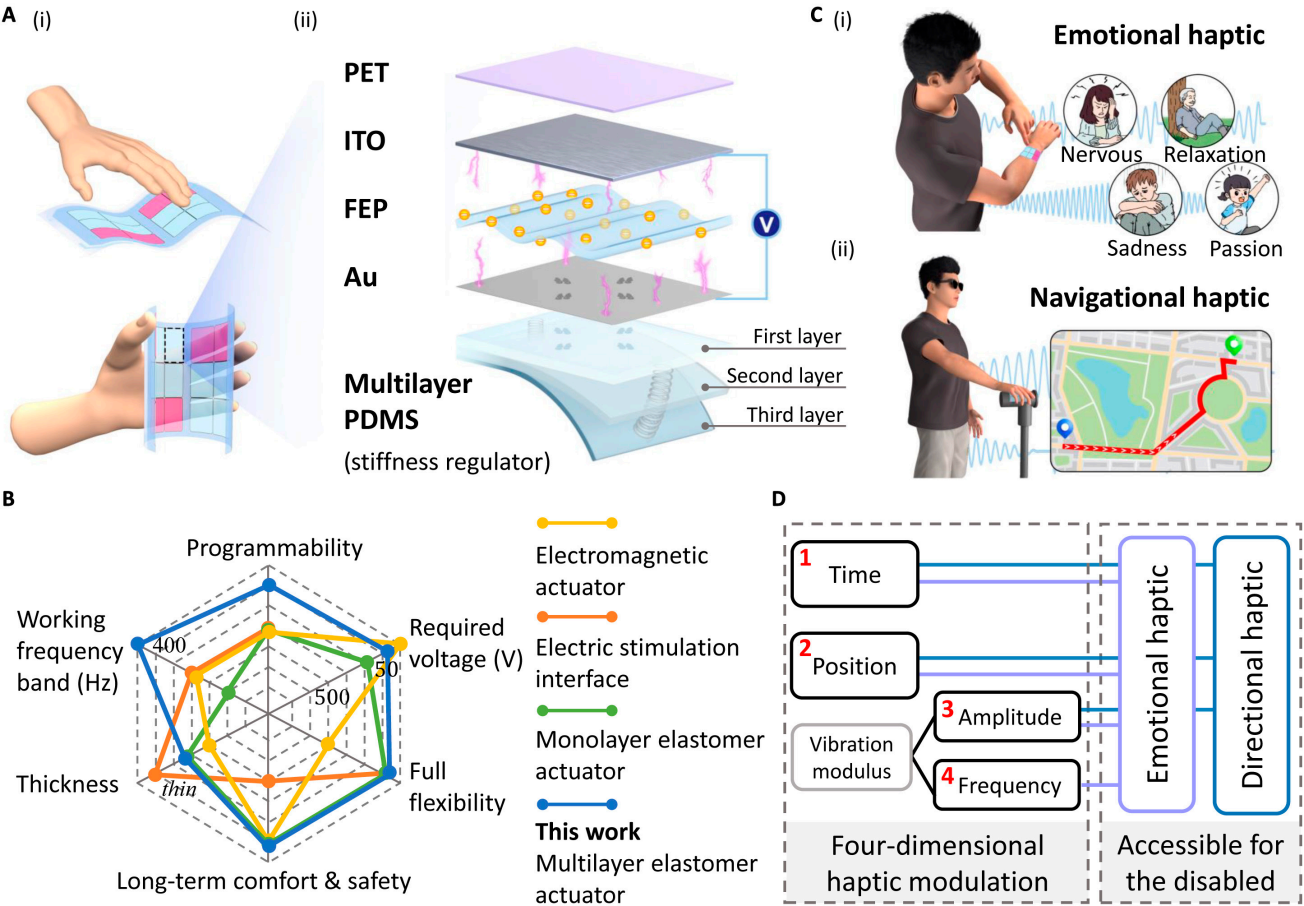
The 4D programmable and low-voltage haptic interface based on elastomer actuators. (A) (i) Concept of a flexible haptic interface based on the low-voltage-driven elastomer actuators for human–machine interaction. (ii) Structure of the actuator prototype, including a multilayer elastomer acting as a stiffness regulator, a charged electret film, 2 electrode layers, and an insulating layer, and the schematic illustration of the actuation mechanism. (B) Performance comparison across 6 dimensions with other reported haptic interfaces. (C) Haptic interface (i) integrated with the skin on a human arm for emotional Braille application and (ii) incorporated into a cane for blind users to facilitate multidirectional haptic navigation. (D) Overview of 4D haptic modulation principles for enhancing emotional and navigational haptic feedback.

Computational studies and experimental measurements demonstrate excellent operational stability and output characteristics. The actuator consists of a 5-layer sandwich structure including a flexible film electrode, a multilayer polymer elastomer electrode, and an electret polymer as the dielectric layer (Fig. 1A). This structure benefits from multilayer PDMS elastomers with variable-stiffness and high-temperature charged electret films, achieving several key features: (a) ultra-low voltage actuation, capable of generating perceptible haptic feedback forces at as low as 5-V actuation; (b) high gain (mN/V), with a gain of 1.06 mN/V at 200-V actuation, and 0.98 mN/V at 35-V actuation; (c) wide frequency bandwidth in the range of 50 to 450 Hz, covering most frequency spectrum of manual sensitivity; (d) an electrostatic charge decay rate of less than 6% after 96 h and then stabilizes, which helps the actuator maintain high electrostatic potential through the electret film, thereby enhancing long-term comfort and safety through low-voltage actuation. Compared to interfaces based on other principles, the proposed low-voltage-driven haptic interface can not only reduce the potential risk of electric shock and electrical breakdown but also contribute to lower energy consumption, and less aging of the dielectric material, thus extending its service life (Fig. 1B). Additionally, since the output force is generated through vertical motion, the size of the actuator can be customized by cutting outside of the microstructure area to meet the spatial requirements of various application scenarios.

With the integration of driving strategy design and array expansion, the haptic interface in this work offers a unique 4-dimensional (4D) haptic programming capability, encompassing time, position, amplitude, and frequency. As illustrated in Fig. 1C, our elastomer actuators enable the interface to (a) simulate fundamental human emotional experiences from a haptic perspective, naturally inducing emotional responses and providing immersive sensations for individuals with visual or hearing impairments, and (b) generate continuous signals across multiple points, recreating a real, arrow-like directional flow along the skin to efficiently fulfill navigational needs of visually impaired individuals without requiring a complex learning process (Fig. 1D). This work lays the foundation for developing safe, fully flexible, and compliant haptic interactive electronic skin in the future.

## Results

### Design and structure of the low-voltage elastomer actuators

**Fig. 2.**
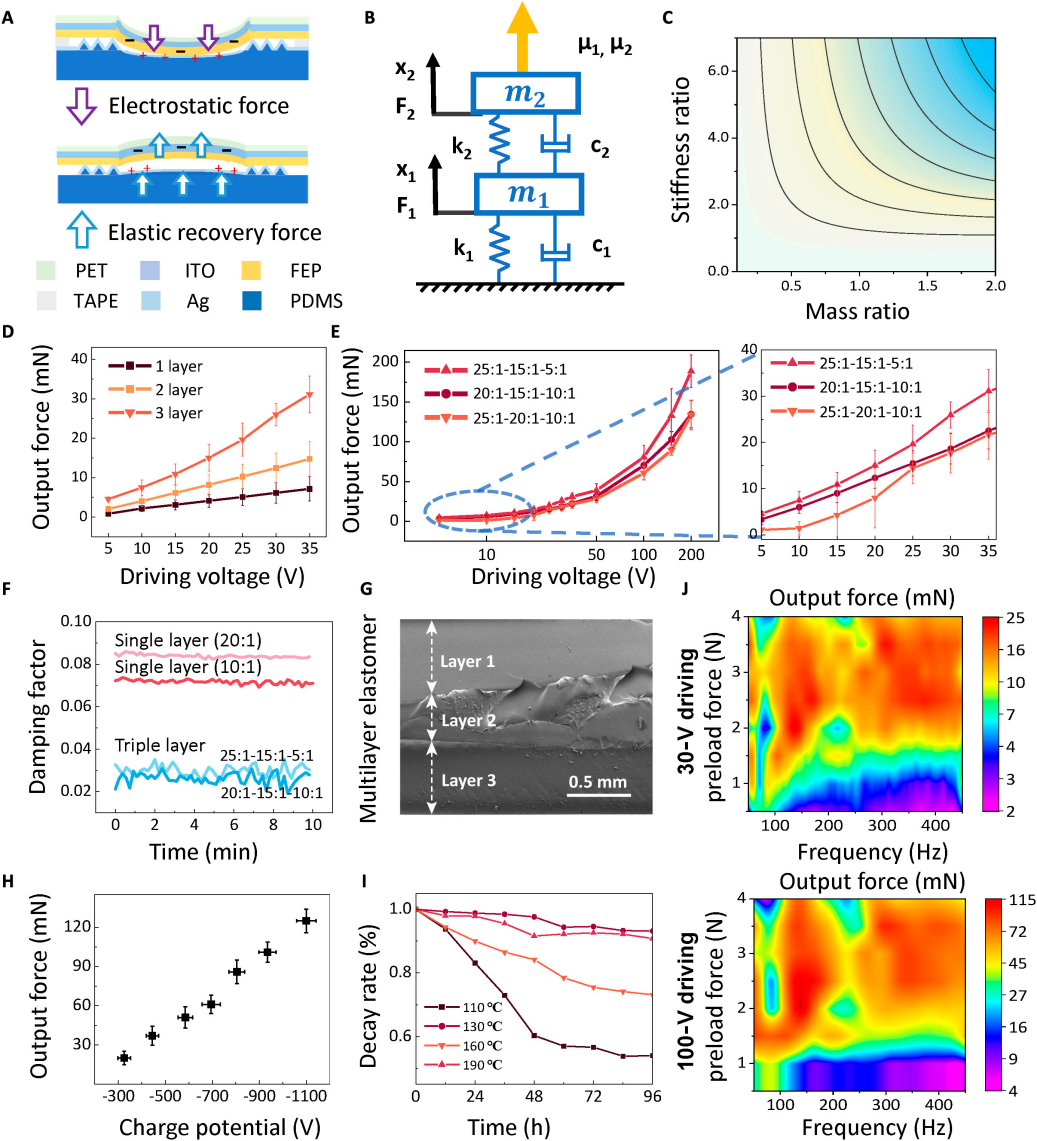
Characterization of the low-voltage-driven elastomer actuator. (A) Cross-sectional view of the vibration of the actuator. (B) Schematic model of the bilayer elastomer as 2 coupled second-order spring oscillators in series. (C) The theoretical results examine the amplitude ratio ($\mu_{1}$) of the first intrinsic frequency in relation to the stiffness ratio ($\tau =k_{1}/k_{2})$ and mass ratio ($\sigma =m_{1}/m_{2}$) between the 2 layers of elastomer. (D) Measured output force of a 1-layer, 2-layer, and 3-layer actuator under an applied driving voltages from 5 to 35 V. (E) Measured output force of three 3-layer elastomer actuator configurations with varying stiffness ratios and fixed mass ratios under driving voltages ranging from 5 to 200 V. (F) Measured damping factors for single-layer and triple-layer elastomers. (G) A cross-sectional SEM photo of the multilayer elastomer, with the 3 layers visible and solidified cured as an inseparable unit. (H) Measured output force of a prototype actuator with different charged electret film surface potentials under an applied peak-to-peak voltage of 100 V. (I) Decay of surface potential of charged electret film over time at different charging temperatures. (J) Measured output force of the selected actuator under a peak-to-peak driving voltage of 30 and 100 V using a 130 °C high-temperature charged FEP electret film, with frequency sweeps from 50 to 450 Hz.

The cross-sectional view of the actuator is shown in Fig. 2A. The top flexible film electrode layer [indium tin oxide (ITO)] is sputtered onto the polyethylene terephthalate (PET). Insulated touch is applied from one side of the PET when interacting with a human hand. The corona-charged electret film fluorinated ethylene propylene (FEP) stores a large amount of charge internally, and its high static potential enhances the strength of the electrostatic field between the top and bottom electrode plates. The excellent mechanical properties and thermal stability of FEP films, as well as their low cost, make them ideal choice for dielectric layers. The bottom elastomer electrode is a 1.6-mm-thick PDMS film layer coated with a 190-nm thick gold (Au) electrode through evaporation. The surface of the elastomer electrode layer contains 4 symmetrical groups of microstructures (Fig. S1), which can be adapted to various touch pressures of the human hand.

Initially, the surface microstructure prevents the film electrode layer from collapsing onto the elastomer electrode layer under the preloading force exerted by a human hand. This interaction compresses the film electrode layer, and the surface microstructure’s design is for the actuator’s resistance to this pressure. Three microstructure shapes are explored here under the same range of pressures (Fig. S2). Our findings indicate that the prismatic structure has a moderate deformation, which helps maintain a gap between the thin-film electrode layer and the elastomer electrode layer. Different sizes of prismatic microstructures (0.5, 1.0, and 1.5 mm) are also experimented, and they display consistent frequency response characteristics and repeatability (Figs. S3 to S5). A tri-prism shape with a 1 × 1 mm square base and 0.5 mm height is selected because it produces a higher output force under typical human touch pressures.

When a sinusoidal AC voltage is applied, it triggers the electrostatic force between the actuator’s flexible film electrode layer and the elastomer electrode layer, causing them to compress inward. The compression and deformation of the 2 electrode layers reach their maximum when the voltage reaches its peak. As the voltage decreases, the electrostatic force lessens, allowing the elastomer’s elastic force to take over and expand the film upward. This alternating sinusoidal AC voltage cycle enables the actuator to move continuously in the vertical direction, providing haptic feedback force to the user’s hand. The actuator’s working principle, as shown in Fig. S6, relies on electrostatic forces for compression and elastic forces for expansion (Fig. 2A). Thus, the low-voltage-driven and high-output force design of the actuator can be achieved by enhancing both the elastic force and electrostatic force, which represent mechanical and electrical aspects, respectively.

To further understand how mechanical properties affect the performance of elastomer actuators with varying stiffness from different cross-linking densities, a corresponding physical model was constructed to quantify the elastic force within the multilayer PDMS elastomer. Given that the polymer elastomer functions as a spring-like component within the system, its physical behavior can be theoretically modeled using a second-order spring oscillator [26]. Here, we analyze a bilayer elastomer electrode layer as 2 coupled second-order spring oscillators in series (Fig. 2B), comparing it to a single layer to assess the elastic force under a fixed electric field. The mechanical vibration of the system can be divided into homogeneous solutions (free motion) and particular solutions (forced motion). We control the parameters in the particular solution to increase the elastic recovery force. The detailed derivation, shown in Figs. S7 and S8, quantifies the bilayer elastomer’s vibration function using 2 parameters: (a) the stiffness ratio ($\tau =\frac{k_{1}}{k_{2}})$ and (b) the mass ratio ($\sigma =\frac{m_{1}}{m_{2}}$) between the 2 elastomer layers. Due to the thickness constraints and the curing requirements of PDMS mixtures, we restrict the range of stiffness ratios (0≤τ≤7) and mass ratios (0≤σ≤2). This is because PDMS mixtures with too low cross-linking density are challenging to cure and increase the elastomer’s viscosity, while mixtures with insufficient weight tend to form elastomer layers with non-uniformity, as different cross-linking mixtures interpenetrate. Thus, we avoid setting higher stiffness and mass ratios. As shown in Fig. 2C, a stiffness ratio of 1 means a single-layer elastomer without stiffness modulation. The theoretical result reveals that the bilayer elastomer’s elastic force scales with the amplitude ratio ($\mu_{1}$) of the first intrinsic frequency (Figs. S7 and S8), which can be optimized in 2 aspects: (a) higher mass ratio and (b) higher stiffness ratio. This model analysis provides direction for enhancing elastomer layer performance.

A series of experiments are conducted using elastomers with various configurations. Figure 2D shows the measured output force responses of a 3 × 3 cm^2^ square actuator with 1, 2, and 3 elastomer layers, all with a fixed total thickness, under driving voltages up to 35 V and a 90-g load to simulate human touch pressure. The results indicate that multilayer elastomer actuators generate significantly higher output forces than single-layer configurations. For instance, the 3-layer elastomer actuator produces an average output force of 4.6 mN at a minimum driving voltage of 5 V, reaching a maximum force of 34.4 mN at 35 V. In contrast, the single-layer actuator achieves only 7.9 mN under the same 35-V driving condition. Further investigation on stiffness ratios was performed by testing 3 different stiffness configurations in a 3-layer elastomer actuator, with a fixed mass for each elastomer layer in each sample but varied stiffness ratios. For each layer of the elastomers, stiffness is governed by the cross-linking ratio, with higher ratios corresponding to lower stiffness. For multilayer structures, the stiffness ratio refers to the relative difference in stiffness among the constituent layers. Given that all samples share the same structural design, the observed differences in stiffness are primarily attributed to variations in the intrinsic material properties of the elastomers, rather than geometric or mass-related factors. The measured Young’s modulus and Poisson’s ratio for elastomers with different cross-linking ratios (Table S1 and Fig. S9) effectively characterize their mechanical properties. The results show that increasing the cross-linking ratio leads to a corresponding decrease in Young’s modulus, while the Poisson’s ratio remains nearly constant. This trend further suggests that a lower ethoxysilane content, associated with a higher cross-linking ratio, effectively reduces the stiffness of the PDMS elastomer, resulting in a softer material. Figure 2E shows the output force of these actuators across a voltage range of 5 to 200 V, demonstrating that a higher overall stiffness ratio results in greater output forces. The output force of the one-layer elastomer and optimized-stiffness-ratio multilayer elastomer actuator under applied voltages ranging from 5 to 200 V is presented in Fig. S10. To provide a comprehensive view of the tested configurations, a total of 10 scenarios, including 1-layer, 2-layer, and 3-layer designs, are detailed in the Supplementary Materials (Table S2 and Fig. S11). These combinations were tested to investigate the mechanical effects of the layered structure. In the 3-layer elastomer configurations, the middle layer was fixed at a 15:1 cross-linking ratio, while the first and third layers are varied in stiffness using different step changes. These are then combined in different schemes to create 4 distinct scenarios, enabling a more structured examination of the influence of stiffness gradients within the multilayer architecture. Figure 2F shows that 3-layer elastomers with increasing cross-linking density in each layer significantly reduce damping compared to the single-layer configuration, highlighting the effectiveness of the multilayer design in achieving higher elastic recovering force under the same electrostatic field. Consequently, the multilayer elastomers act as effective stiffness regulators, lowering the actuator’s required driving voltage and enhancing vibratory motion from a mechanical perspective. A cross-sectional scanning electron microscope (SEM) image of the 3-layer elastomer is provided in Fig. 2G, offering visual insight into our layered structure.

The electret film FEP is used here as the dielectric layer in the actuator to improve the electrostatic force from an electrical perspective. The corona charging process allows the electret film to store a large amount of static charge inside, which gives the film a high static potential energy and superimposes the electric field between 2 electrode layers (Figs. S12 and S13). The amount of charge stored inside the electret film and the storage time determine the effectiveness of the process. When applying a fixed electric field, the actuator’s output force significantly increases with the increase of charged potential (Fig. 2H). The ability of the electret film to maintain static charge over time determines the low-voltage-driven actuator’s lifespan. Heat treatment of the charging process significantly improves the charge storage stability of electret films [34,35] and improves their lifetime to decades or even centuries [36–38]. Thus, the high-temperature corona charging process (Fig. S14) is employed here to assist inward migration of the charge’s gravity center by electret film, helping it maintain high charged potential for a long period. Figure 2I plots the charge decay rate within the unprotected electret film following the heat treatment charging process at different temperatures. The optimal charging temperature is 130 °C under our experimental conditions.

Based on previous experiments, output forces are measured by selecting the stiffness configuration of 25:1-15:1-5:1 and the high-temperature charged FEP electret film at 130 °C, with peak-to-peak driving voltages of 30 and 100 V across a frequency of 50 to 450 Hz (Fig. 2J). The results demonstrate that the actuator maintains consistent performance under both high and low voltages, delivering nearly uniform output across the wide frequency range and generating sufficient output force under preload forces from 1 to 4 N.

### System integration and 4D programmable haptic feedback

**Fig. 3.**
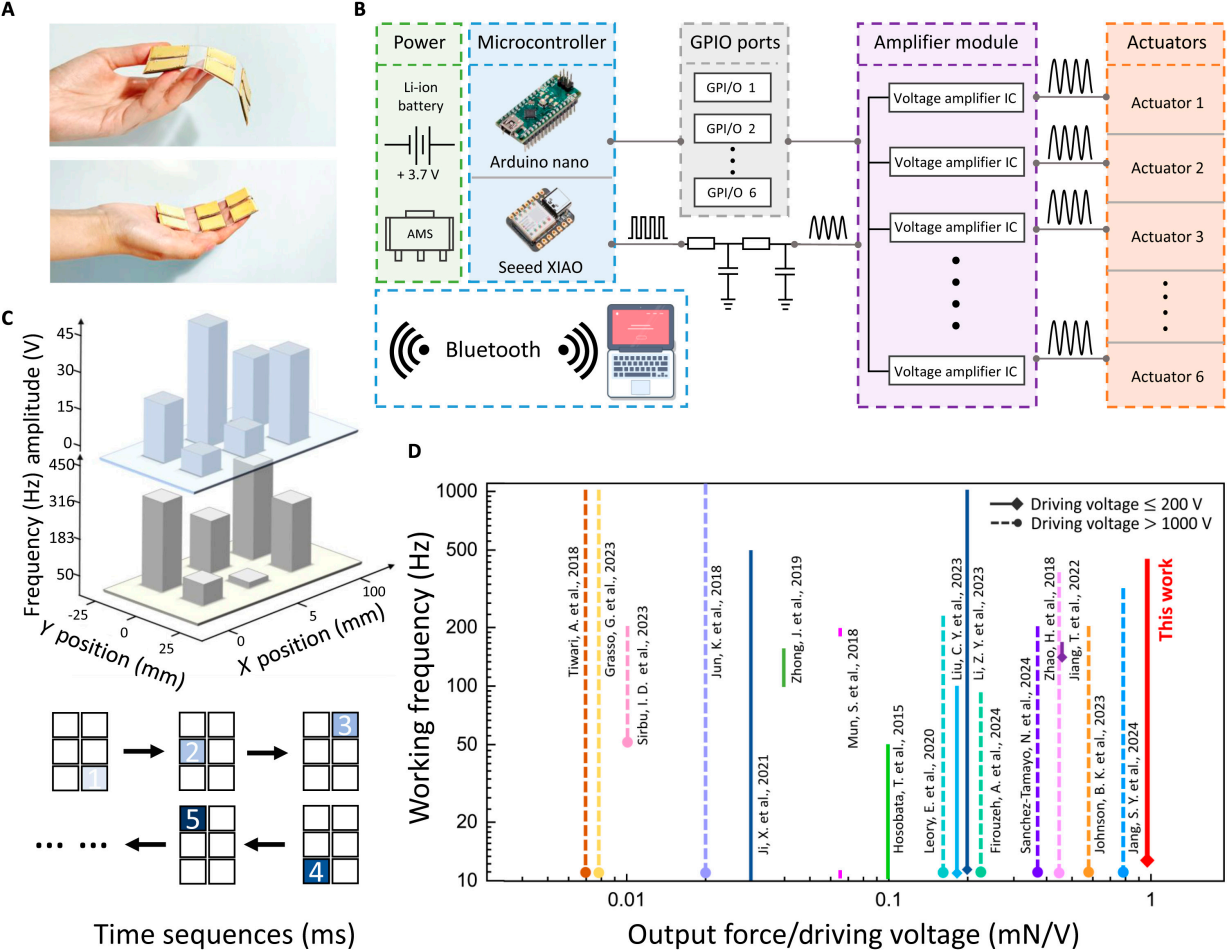
Programming principles and evaluation of the output performance. (A) Optical image of arrayed flexible haptic interface. (B) System integration and circuit diagram for the actuator array. (C) Schematic of the actuator’s 4D (amplitude, frequency, time, position) programming method. (D) Comparisons of output force per applied voltage and working frequency range of other reported flexible actuators.

The feature that low-voltage-driven actuators do not rely on a bulky driving device enables the construction of the arrayed haptic interface and enhances their applicability in human interaction. Here, the actuator units can be freely expanded into any number of array designs. To integrate with the Braille structure for disability assistance, we developed a flexible haptic interface (Fig. 3A and Fig. S15) consisting of a 3 × 2 array of low-voltage actuators. The operational organization and wireless communication with an external control system are depicted in Fig. 3B. Driving signals with controllable frequency (generated by the waveform generator Seeed XIAO) are processed by a second-order low-pass filter as the common input for 6 drivers (DRV 8662). The microcontroller unit (Arduino Nano) responds to Bluetooth wireless messages and provides the operational logic of these actuators through 6 digital potentiometers (X9C104). The amplified voltages with modulation are then generated. This allows different modes in the remote upper computer to be wirelessly streamed into 6 actuators, providing users with different haptic experiences. Each actuator within the interface can be independently actuated to generate haptic feedback, allowing modulation across 4 dimensions (Fig. 3C): time, position, amplitude, and frequency. Testing with 10 volunteers involves continuous point discrimination touch at different drive voltages and vibration durations (Fig. S16). Results confirm that the arrayed actuators are discernible at low drive voltages and short durations. Based on this 4D modulation, the haptic interface is capable of delivering emotional and navigational haptic feedback, mimicking the emotional nuances found in speech and providing directional signals that simulate real flows.

The output force per applied voltage and working frequency of the proposed actuator is compared with those of other state-of-the-art flexible actuators [20,21,26,39–52] in Fig. 3D. Additionally, key differences in materials and structural design between our actuator and other elastomer-based actuators are summarized in Table S3. Due to the introduction of the stiffness control strategy for multilayer elastomers, our actuator achieves the highest relative output force per applied voltage among reported actuators under both high-voltage and low-voltage driving conditions. We also obtain a programmable frequency range of nearly 400 Hz, enabling multimodal haptic output while reducing high-voltage risks and the need for bulky drive systems. Compared to haptic feedback interfaces with other working principles, our interface provides unique advantages for multifunctional haptic interaction interfaces, including wireless operation, rapid response time, wide working frequency range, exceptional programmability, and suitability for safe, comfortable, and long-term use (Fig. 1B).

### Emotional Braille interaction system using haptic interfaces

**Fig. 4.**
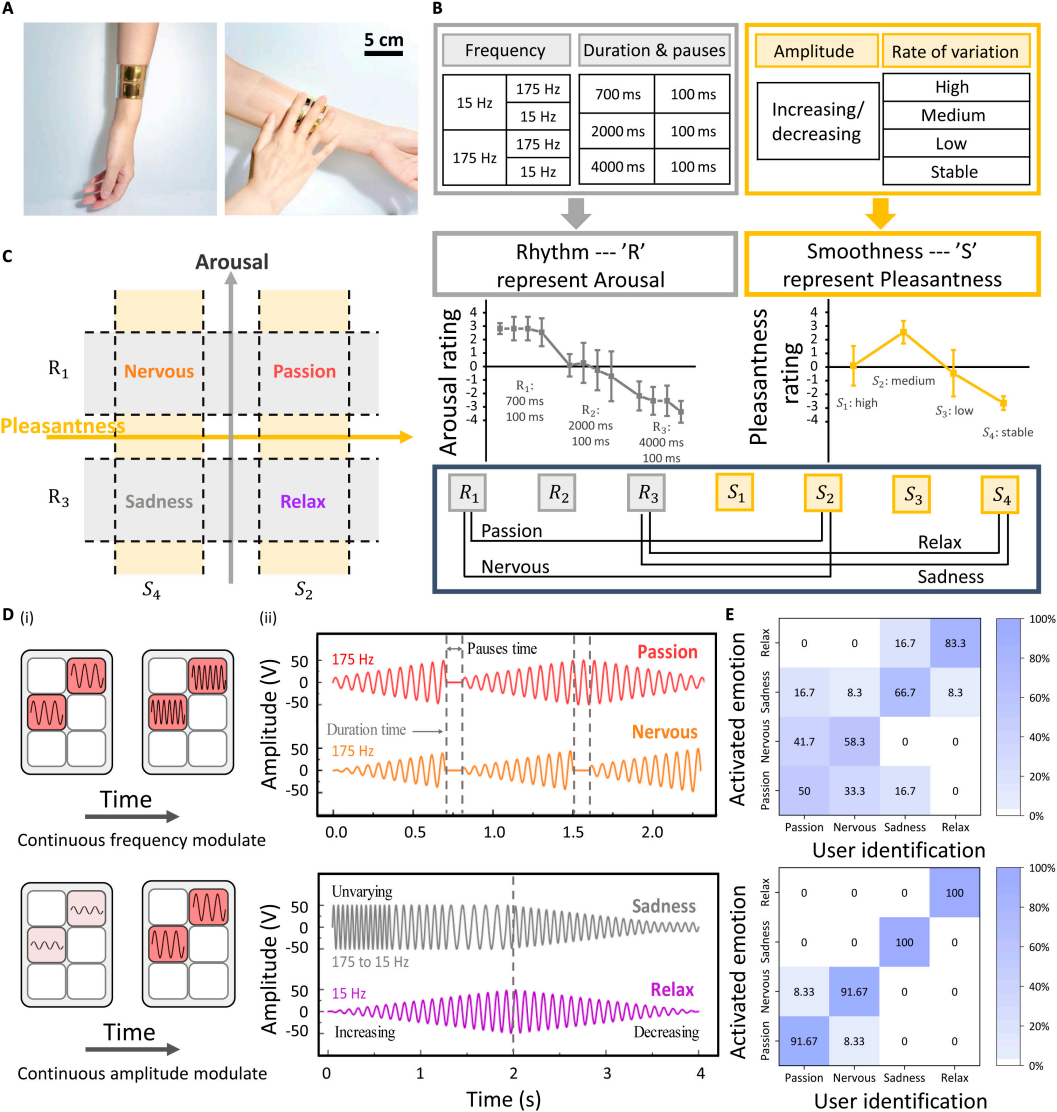
Emotional programming design for haptic interfaces. (A) Schematic of integration and interaction with the human arm in the haptic interface. (B) Design for arousal and pleasantness. The combination of frequency, vibration duration, and pause time is modulated as rhythm “R” for arousal; the combination of vibration amplitude and its rate of variation is modulated as smoothness “S” for pleasantness. (C) Emotional haptic feedback coordinate chart showing 4 emotion archetypes (“Passion”, “Nervous”, “Sadness”, “Relax”), mapped with arousal as the vertical axis and pleasantness as the horizontal axis. (D) Schematic of the (i) frequency–amplitude modulation and (ii) actuation signals of 4 emotional archetypes. (E) Confusion matrix for 12 volunteers: Percentages represent the emotions correctly identified by users for the 4 stimuli depicted in (D); the diagonal line percentages (i.e., correct responses) range (i) from 50% to 83.3% without learning mode and (ii) from 91.67% to 100% with a learning mode.

The haptic interface (Fig. 4A) is designed to match the structure of the Braille system to output haptic Braille information driven through the wireless Bluetooth system. Leveraging 4D programmability—frequency, amplitude, time, and position—it facilitates nuanced emotional haptic interactions similar to musical expressions. Just as music is composed of elements like frequency, amplitude, and melody, this haptic system leverages similar variables to create rich sensations that mimic emotional cues similar to those found in music.

We observe the haptic interface’s ability for emotional communication by associating certain parameters of haptic feedback with rhythm (“R”) and smoothness (“S”). The driving frequency, vibration duration, and pause time of the actuator can correspond to the rhythm, which is used to simulate arousal here, while the driving amplitude and its rate of change can correspond to smoothness, which is used to simulate pleasantness. In Fig. 4B, we examine 3 different vibration durations and pause times, each with 4 directions of frequency flow. Testing results from 15 volunteers indicate that the vibration duration and pause time play a key role in simulating arousal. We firstly assess the effect of a single-frequency signal at the same duration and pause time on volunteers’ emotions, finding that high-frequency vibrations increase arousal, whereas low-frequency vibrations result in the lowest arousal (Fig. S17). In the rhythm test here (Fig. 4B), frequency flow toward lower values is found to induce lower arousal, especially at longer vibration duration and pause times. However, shorter duration and pause times with low frequencies still produce relatively higher arousal. These findings underscore that although lower frequencies tend to reduce arousal, the duration and pause times have a more dominant influence. Regarding smoothness, as test with various amplitude variations and rates of change (including increasing and decreasing), continuous and gradual variations in vibratory force—either a slow rise followed by a fall or a steady constant—tend to induce a higher pleasantness. Rapid, unidirectional changes in force are generally perceived as unpleasant. Based on these findings, we obtain 3 rhythm programming modes (R1, R2, and R3) for different arousal levels, and 4 smoothness programming modes (S1, S2, S3, and S4) for different levels of pleasantness. We utilize these modes to plot fundamental emotions on 2D coordinates (Fig. 4C), with pleasantness on the horizontal axis and arousal on the vertical axis [53,54]. This mapping allows us to associate higher arousal and pleasantness with “Passion”, while lower levels correspond to “Nervous”, “Sadness”, and “Relax”, respectively. This framework aids in the precise modulation of haptic feedback to evoke targeted emotional responses.

Figure 4D illustrates the amplitude–frequency modulation strategies and driving signals for the 4 fundamental emotions. Use the letter “I” in Braille as an example [Fig. 4D (i)], the continuous frequency modulation highlights how the driving frequency changes over time, while the continuous amplitude modulation demonstrates how the driving voltage can be tailored over time. By applying this strategy to different words placed at various spatial locations on the interface, we can effectively convey the 4 emotions through haptic Braille. As shown in Fig. 4D (ii), “Passion” and “Nervous” both utilize a fast rhythm with a vibration time of 700 ms and a pause time of 100 ms. However, “Passion” features smoother amplitude changes, while “Nervous” is characterized by a unidirectional amplitude increase. In contrast, Sadness and Relaxation are associated with slower-paced haptic feedback, with vibration times of 4,000 ms and pause times of 100 ms. Sadness involves a unidirectional amplitude decrease, whereas Relaxation includes a gradual and continuous amplitude increase and decrease. As illustrated in Fig. S18, we convey the phrase “I love U” through these 4 fundamental emotional driving signals on the arrayed haptic feedback interface, where each letter vibrates sequentially. Figure 4E presents the confusion matrix for the haptic feedback test of the Braille text “I love U” expressed with 4 different emotions. The experiment includes 12 volunteers who participate in 2 rounds of testing. In the first round, without any learning mode, participants interact with the interface to determine the emotions conveyed by the interface based on their initial impressions, achieving an average accuracy of 64.6%. This result indicates that the haptic signals driven by emotions are inherently effective at evoking distinct emotional responses. In the second round of testing, after a learning phase, the accuracy in recognizing different emotions increased significantly, with accuracy ranging from 91.67% to 100%.

### Real touch flow sensations in navigation systems using haptic interfaces

**Fig. 5.**
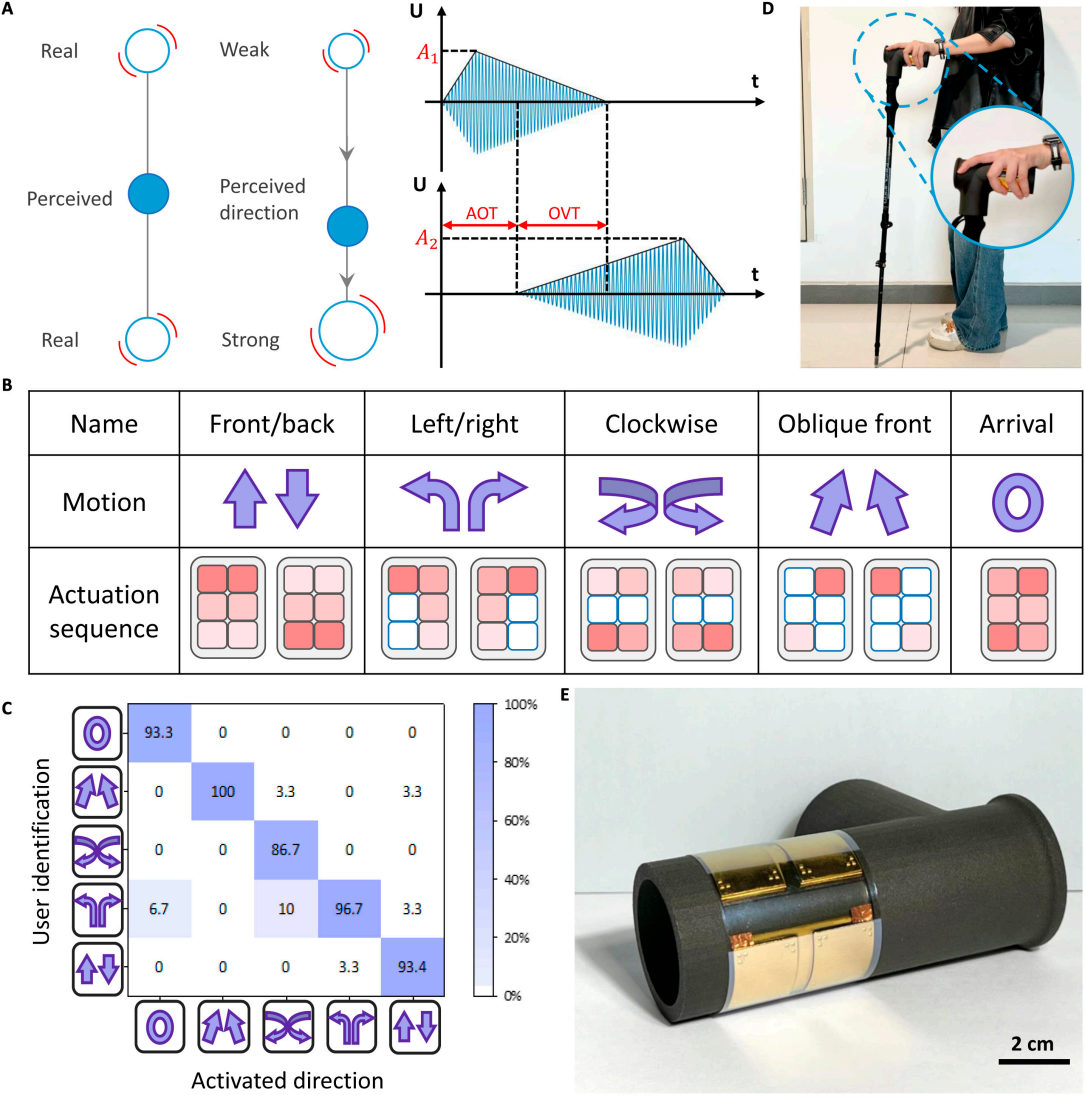
Demonstration of real touch flow navigation system. (A) Schematic of the 2-point haptic illusion about touch flow sensations and actuation method. (B) Direction patterns that displayed to volunteers during tests of the haptic navigation system. (C) Confusion matrix for 15 volunteers: Percentages indicate the patterns recognized by users for 5 stimuli shown in (B); the percentages in the diagonal line range from 93% to 100%, with an average recognition time of less than 4.3 s, indicating that the real flowing haptic navigation system can be easily and conveniently recognized by the user. (D) A user operates a cane equipped with an integrated haptic interface. (E) Guide cane handle features the integrated haptic interface; Figs. S24 and S25 show the actuating circuitry.

The 4D programmability of the haptic interface introduces new solutions for haptic navigation systems. Here, we present the haptic illusion model of continuous touch flow across human skin [55], where users perceive directional flow between 2 points by modulating vibration timing and amplitude levels (Fig. 5A). Key parameters—including the actuation flow direction (AFD), actuation onset time (AOT), overlap vibration time (OVT), and actuation amplitude (A) between the 2 actuator units—are experimentally characterized with 10 volunteers. Each volunteer places their fingertips on a haptic interface, experiences various drive signals, and selects the most effective flow sensation based on their perception. Detailed design steps and experimental data are presented in the Supplementary Materials (Figs. S19 to S23 and Tables S4 and S5). Results indicate that in our arrayed haptic interface, when the actuation progresses from an initial weak haptic actuator to a stronger one, users experience a more realistic directional touch sensation. The optimized actuation parameters are an AOT of 600 ms, an OVT of 200 ms, and an actuation amplitude ratio of ~1.67 times. We apply these findings to design multidirectional commands for haptic navigation across multiple actuators, as shown in Table S6. Leveraging the arrayed design and haptic illusion model, the interface activates across multiple actuator units, generating a realistic directional haptic flow. Compared to other navigation interfaces that rely on single-mode information and artificially defined sensations, our system allows users to quickly recognize natural haptic directions.

We assess the ability of volunteers to recognize the directional commands from our haptic interface. The sets of the navigation system for 5 different motions—front/back, left/right, clockwise, oblique front, and arrival—and the corresponding actuation sequence are shown in Fig. 5B. Fifteen volunteers are asked to recognize the haptic navigation system after a learning phase. Five different motions with 8 different patterns are randomly displayed to users, and they are asked to identify them. These patterns consisted of 2 to 4 vibration steps, lasting between 2.2 s (oblique front) and 3.4 s (left/right and clockwise). An average recognition time of 4.3 s was observed across all patterns, including the duration of the haptic signal. The confusion matrix in Fig. 5C shows the highest recognition accuracy of 100% in oblique forward and an average recognition accuracy of 94.2% for all patterns. We also find that the “turn left/right” is more likely to be mistaken for “front/back” or “clockwise”, which can be attributed to the same flow segments shared in their vibrational programs.

With the integration of stiffness-variable elastomer and high-temperature charged electret film, this work develops an actuator capable of “low voltage–high output” performance, eliminating the need for bulky actuation devices. As a result, our arrayed haptic interface can be wirelessly actuated and integrated via the customized circuit board into a cane (Fig. S24), as shown in Fig. 5D and E, providing a convenient solution for a broad range of disability-assisted applications.

## Conclusion

We present a low-voltage-driven and highly programmable 4D haptic interface that is created using a multilayer variable-stiffness elastomer process for applying low-damping vibration. By utilizing this elastomer forming approach along with the high-temperature charged electret, our actuator achieves both mechanical and electrical improvements to deliver an immersive haptic experience for the disabled community. Specifically, it (a) generates haptic feedback forces of approximately 4.6 mN at a minimum driving voltage of 5 V; (b) offers a programmable frequency range of 50 to 450 Hz, covering the spectrum of human skin sensitivity; and (c) operates at a driving voltage within 50 V, ensuring long-term safety and comfort by using isolation and reducing the electret’s surface charge decay rate to below 6%. Additionally, we introduce a programming method that links mechanical vibrations on the skin to emotional responses. Participants in an experiment noted, “Touching this section of the program makes me suddenly feel nervous.” This feedback underscores the potential of our haptic interface to evoke specific emotional responses, indicating that this approach could open new avenues for exploring emotional dimensions in human–machine interaction.

In comparison to previous state-of-the-art flexible haptic interfaces, we have made improvements for 2 major limitations: the necessity for bulky, high-voltage driving setup and the monotonous haptic information that lacks differentiation in time and space. Four fundamental emotions and 9 navigation commands are validated here through our device and software program. Future work may involve further refining the resolution of the emotional coordinates to enhance the complexity and subtlety of the haptic simulation. To evaluate real-world applicability, particularly for visually impaired users, collaborations with organizations serving the blind community are being pursued, alongside plans for targeted user studies. Furthermore, the multi-stiffness soft elastomer materials employed here can also function as a packaging material for different sensor and actuator structures. Combined with hydrogel electronic skin, this technology is expected to facilitate a customizable pixelated interface for emotional management in wearable devices. Interdisciplinary collaboration with experts in neuroscience and psychology could further extend the technology’s application in areas such as depression treatment, personalized rehabilitation, educational tools, and virtual reality—contributing toward the development of a more inclusive, barrier-free society.

## Materials and Methods

### Fabrication of the multilayer elastomer electrode

A 3D-printed mold was designed by SolidWorks and printed using photosensitive resin (Somos10122). The standard features on these molds were a set of 4 symmetrical tri-prismatic grooves, each tri-prism having a square base with a side length of 1 mm and a height of 1 mm. The overall shape of the molds was that of a groove, which was 1.6 mm deep, allowing good control of the thickness of the PDMS elastomer layer. PDMS mixtures (5:1, 10:1, 15:1, and 25:1) were mixed and stirred. The multilayer PDMS elastomers should be poured into the mold by layers. A 25:1 PDMS mixture was poured first and vacuumed for 10 min. After the surface had been flattened, a 15:1 PDMS mixture was poured evenly in a “Z” pattern and vacuumed for 10 min. Then, a 5:1 PDMS mixture was poured in the same steps. After all 3 layers of PDMS mixtures were poured in, air bubbles were vacuum-removed for 30 min and cured for 24 h at 45 °C (Fig. S26).

### Fabrication of the actuators

Metals were vaporized on multilayer PDMS elastomers using electron beam vaporization as the elastomer electrode layer of the actuator. Chromium electrodes with a thickness of 5 nm were first vaporized on a multilayer elastomer to ensure strong adhesion of the electrode layer, and then Au electrodes with a thickness of 190 nm were vaporized as the conductive layer. The 90-nm ITO is sputtered onto the 125-μm PET substrate (30 to 40 ohm sq^−1^, South China Xiangcheng Technology Co. Ltd.) as the flexible electrode film for the actuator. The FEP was treated with a high-temperature corona charging process (Fig. S26).

### Fabrication of arrayed haptic interface

The haptic interface was made of 3 × 2 actuators according to the standard model of Braille characters. In the arrayed haptic interface, the flexible film electrode layer was prepared in the same way as the actuator unit. The multilayer elastomer electrode layer was selectively masked with PI (polyimide) tape before E-beam evaporation to ensure that the 6 units were differentiated.

### High-temperature corona process

The corona charging system was composed of a high-voltage power source (DW-N303-1ACH2, Dongwen, China), a corona needle, a heating plate, and a ground electrode. First, the heating plate was opened until it reaches 130 °C, and the FEP was placed at a distance of 4 cm below the tip of the charging needle. After −20-kV, 8-min charging, the heating plate was turned off and the high voltage was turned up to −25 kV. Charging was stopped until the heating plate returns to room temperature. The typical charging time was 1 h.

### Electrical characterization

The static potential on the surface of the charged FEP film was measured at room temperature by the electrostatic meter (model 347 Trek), with the probe of the electrostatic meter about 3 mm from the surface of the sample. The points at the center and around the film were measured separately, and the average value was calculated as the static potential of the film.

### Characterization of the actuators

The signal generator (RIGOL DG1022Z) and high-voltage amplifier (ATA-7020, Xi’an Antai Electronic Technology Co. Ltd.) applied frequency- and amplitude-controllable driving voltages to the actuators for the performance characterization, while the customized wireless driving circuit was for users’ experiments. The force sensor (Nano 43, ATI Inc.) was used to characterize the vibration characteristics of actuators under different driving amplitudes, frequencies, and preloads (Fig. S27). Preloads were simulated using weights of different weights. A signal generator was set up to generate a sinusoidal signal with a period of 2.5 s and a frequency range of 50 to 450 Hz during the frequency sweep test. For all the performance characterizations, the resulting data were sampled by Nano 43 (ATI) and then processed by MATLAB 2020.

### Signal processing for vibration output force

The measured output force signals were first band-pass filtered using MATLAB. The mean value of the upper envelope and the lower envelope of the filtered signal was calculated, and the difference was calculated as the vibration output force of the actuator.

## Data Availability

All data required to support the conclusions are presented in the main text and/or the Supplementary Materials.

## Supplementary Materials

Supplementary 1 Figs. S1 to S27 Tables S1 to S6 Notes S1 to S4 Movies S1 and S2
