# Supplementary material for: Emotional and Directional Enabled Programmable Flexible Haptic Interface for Enhanced Cognition in Disabled Community
Source: Research (Wash D C). 2025 Jun 3;8:0714. doi: 10.34133/research.0714 (PMC12133101; doi:10.34133/research.0714)
Supplement: Supplementary 1 — Figs. S1 to S27 Tables S1 to S6 Notes S1 to S4 Movies S1 and S2 [file research.0714.f1.zip › Supplementary Materials-r1-Research.docx]

**SUPPLEMENTARY MATERIALS**

**Emotional and Directional Enabled Programmable Flexible Haptic Interface for Enhanced Cognition in Disabled Community**

*Yuhan Liu^1†^, Liuyang Han^1†^, Siqi Lv^1^, Tao Jiang^1^, Mingkai Duan^1^, Hanyu Guo^1^, Yuzhen Li^1^, Qisen Xie^1^, Yanru Chen^1^, Dongkai Wang^2^, Ziheng Liu^1^, Wenjie Zhang^1^,Yanting Gong^2^, Junwen Zhong^2*^, Xiang Qian^1*^*

1. Tsinghua Shenzhen International Graduate School, Tsinghua University, Shenzhen, 518055, China.
2. Department of Electromechanical Engineering, Centre for Artificial Intelligence and Robotics, University of Macau, Macau, 999078, China.

^†^ These authors contributed equally to this work

* Corresponding author

Email:

[qian.xiang@sz.tsinghua.edu.cn](mailto:qian.xiang@sz.tsinghua.edu.cn)

[junwenzhong@um.edu.mo](mailto:junwenzhong@um.edu.mo)


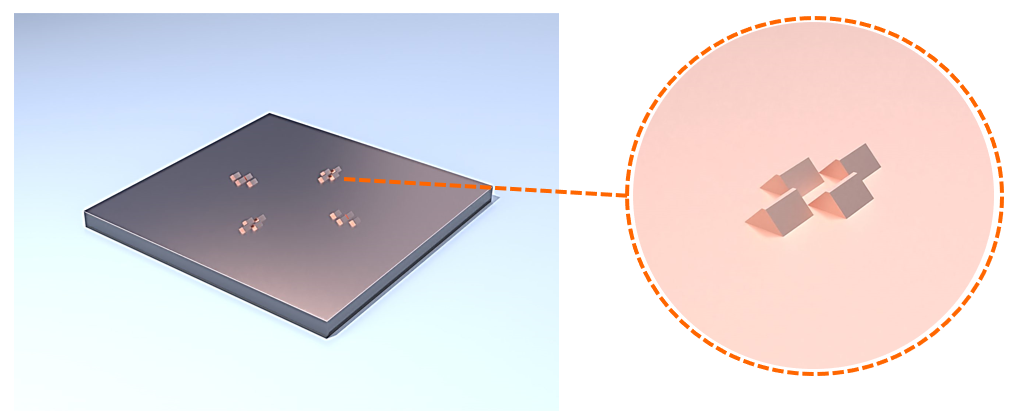


**Fig. S1.** Surface microstructure of the elastomer electrode layer. Microstructure in the shape of a triangular prism. The bottom surface is a square with a side length of 1 mm and a height of 1 mm. Four tri-prisms are grouped and placed symmetrically on the elastomer electrode layer.


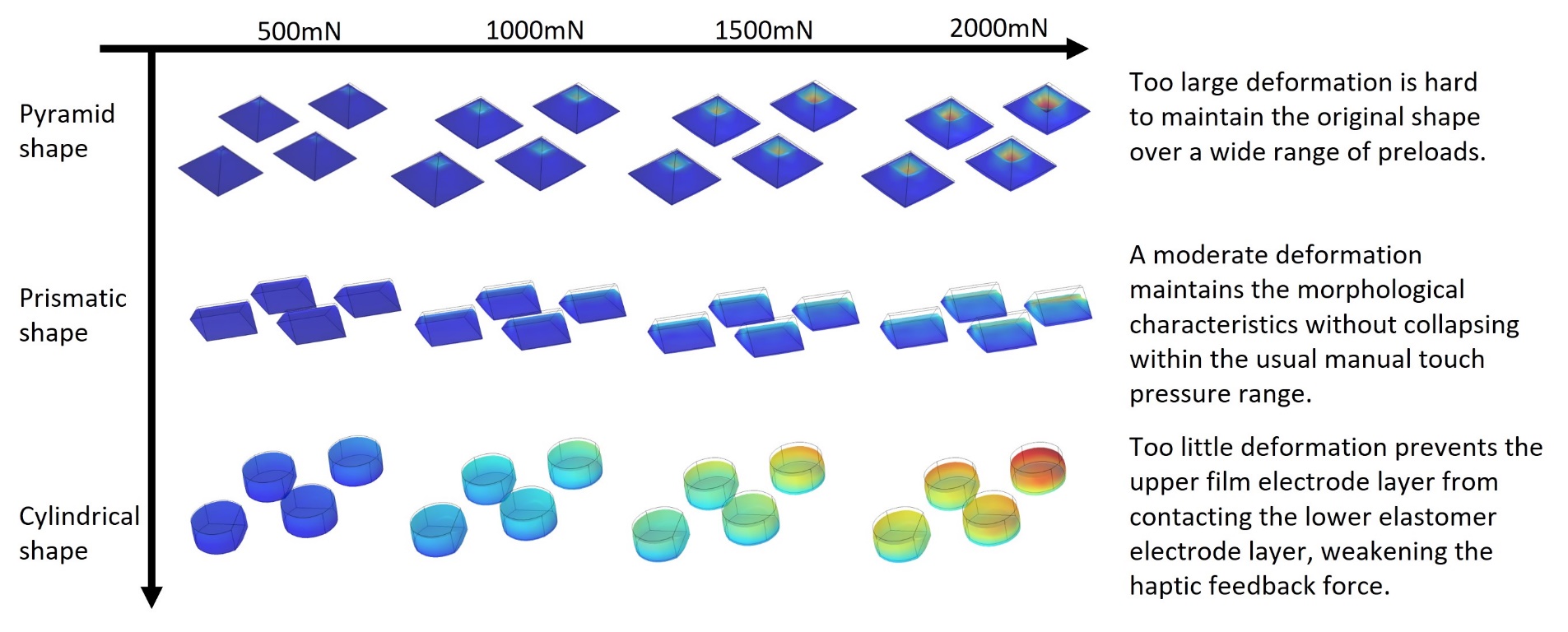


**Fig. S2.** Compression simulation of surface microstructures. Three different shapes of microstructures are simulated. Pressure ranges from 500 to 2000 mN are applied to the actuator surfaces with different shapes as surface microstructures, which are commonly used by human hands for touch.

**Note S1.** Microstructure size of elastomer electrode design. Microstructures of different sizes are designed into the electrode layer of the actuator elastomer. We compare three microstructures with bottom side lengths and heights of 0.5 mm, 1.0 mm, and 1.5 mm, respectively. The actuator output force is measured at the same drive voltage to choose the size that is most suitable for human interaction.


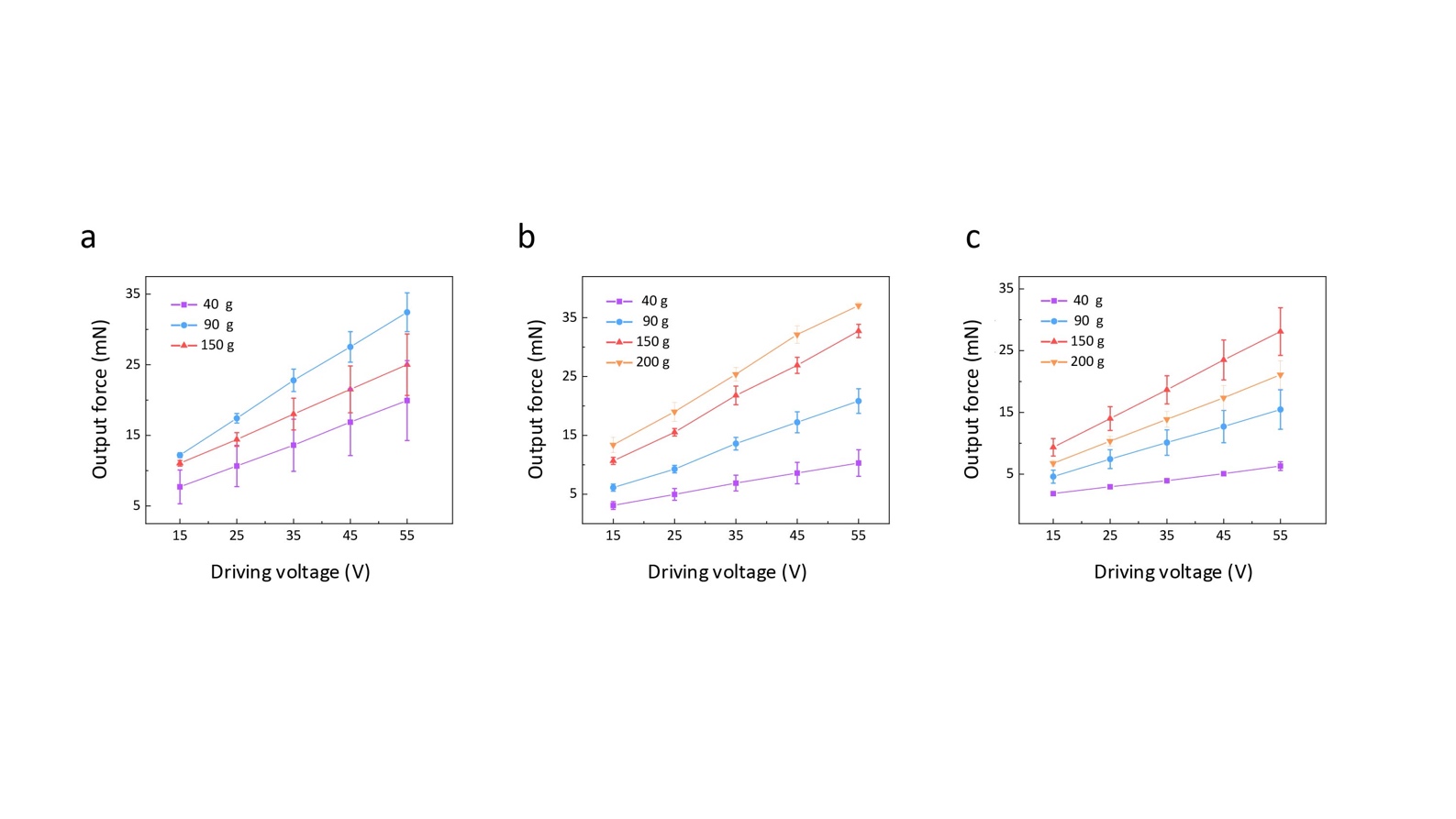


**Fig. S3.** Test Driving voltage – Output force of three different sizes of microstructures. The output response of three different microstructure sizes (0.5 mm, 1.0 mm, 1.5 mm) of the actuator is tested at preload force which use weights (40g, 90g, 150g, 200g) to apply the respective pressures.

Here, the 1.0 mm microstructure size is chosen because it embodies an excellent level of output force at the touch pressures we would expect to see commonly used by a human hand. Comparing the 0.5 mm and 1.5 mm microstructure sizes, the 1.0 mm ones produce a higher output force at the same pressure level. In addition, as the preload force rises, the output force of the 1.0 mm microstructure rises. In contrast, the 0.5 mm and 1.5 mm microstructure actuators reduce the output force at a pressure of 150 g, and 200 g weights, respectively.


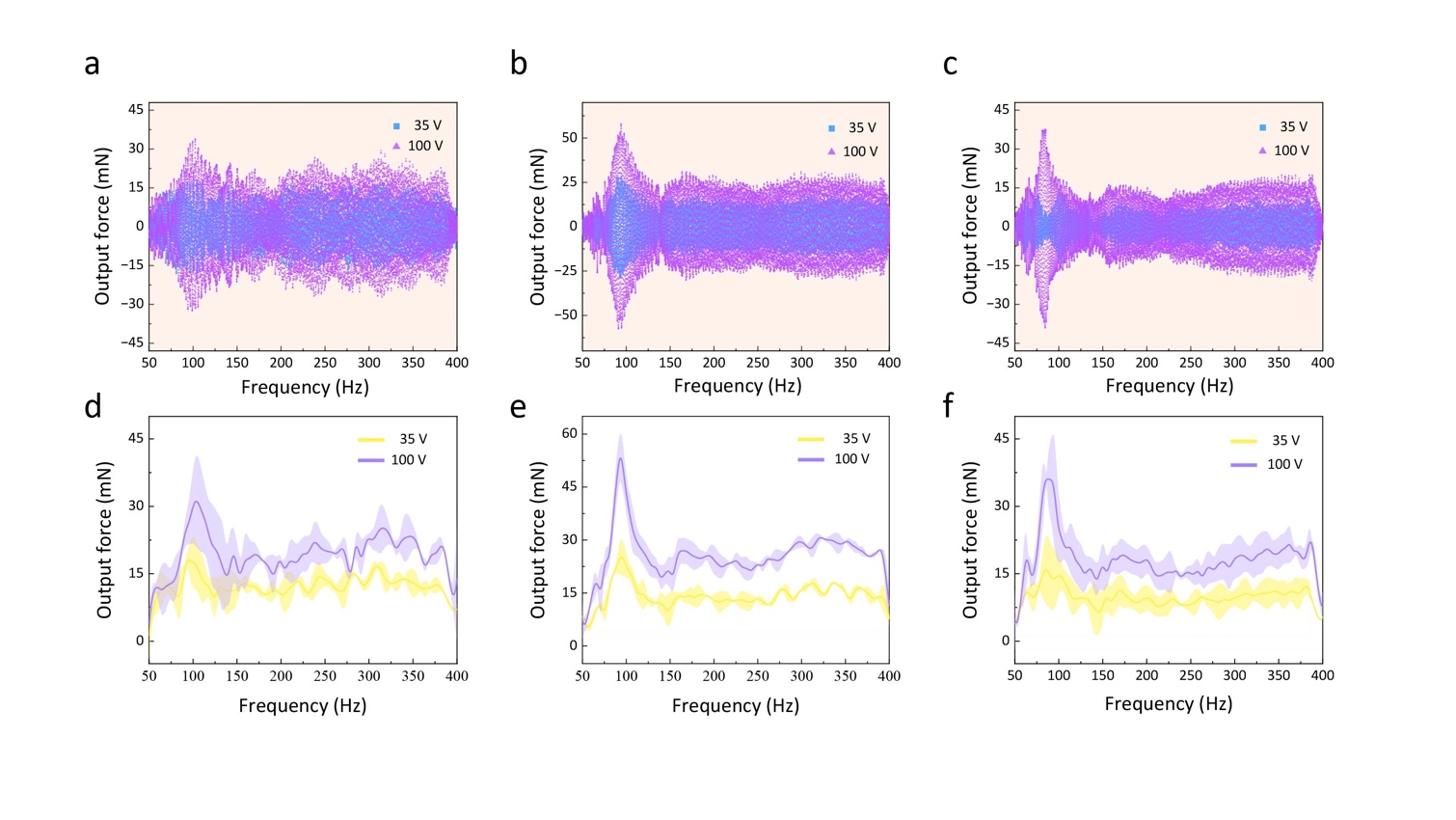


**Fig. S4.** Frequency response of actuators with three microstructure sizes. The output force of the actuator is measured at a frequency of 50 to 450 Hz and an input voltage of 35 V and 200 V. The three sizes of actuators had similar frequency response and error band images, which demonstrates the stability of the actuator output in terms of process preparation.

**Fig. S5.** Comparison of the output force of three sizes of actuators under pressure. The results show that larger-size microstructures allow the actuator's pressure resistance to improve. However, it is not the case that the larger the size of the actuator the higher the pressure resistance. In addition, an oversized microstructure can prevent the upper and lower plates from contacting each other. Electrically, the electrostatic field force is reduced with larger plate spacing. Therefore, a 1.0 mm microstructure is more appropriate here.


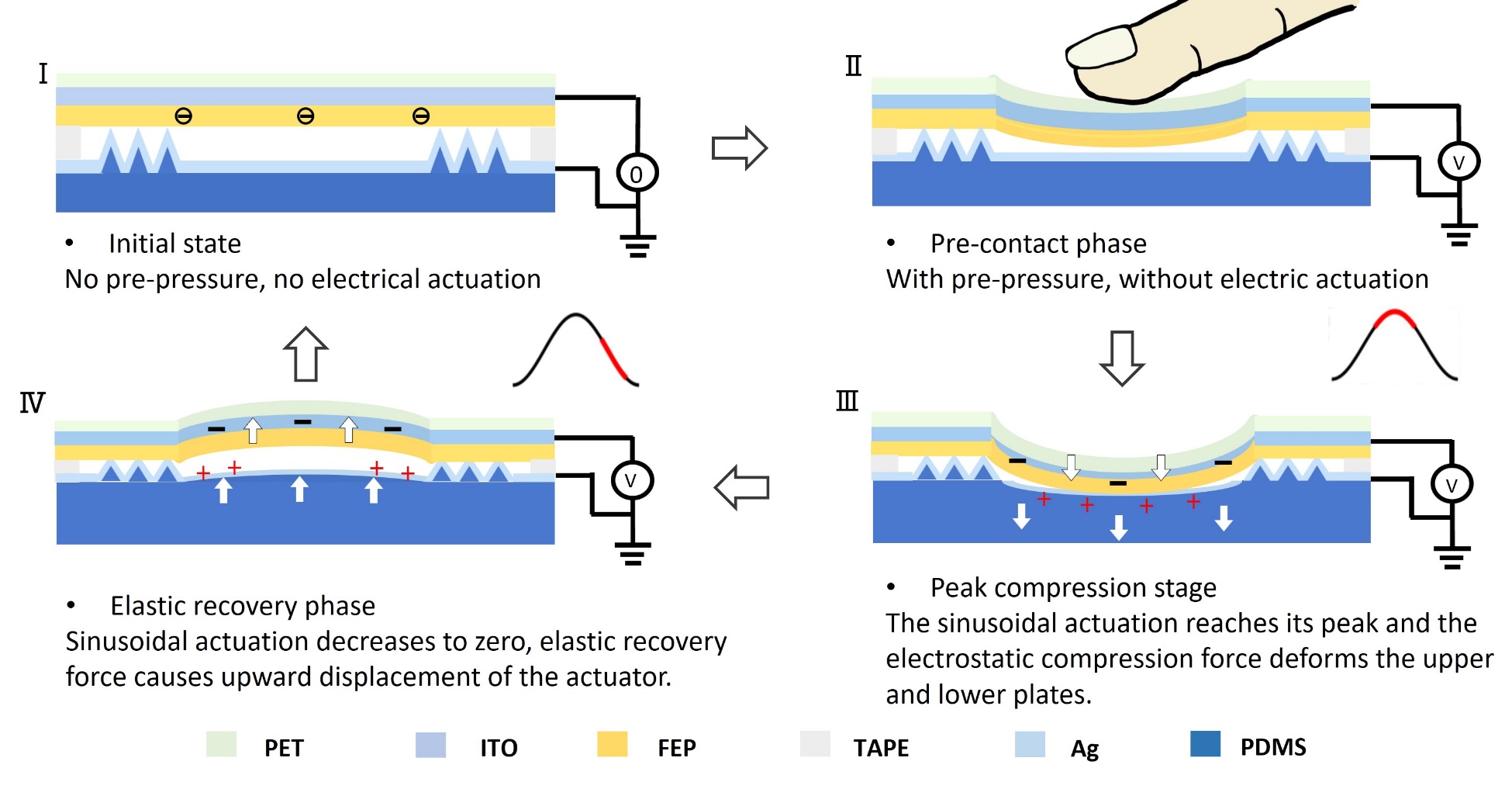


**Fig. S6.** The working principle of the electrostatic actuator. Under a sinusoidal driving voltage, the actuator produces output force and displacement due to the electrostatic force continuously. At the initial state without the external driving voltage and preload force in **Fig. S6-I**, the actuator is balanced both in mechanical and electrical perspective. In **Fig. S6-II**, an external force from the human hand is applied on the actuator diaphragm and deforms downward as shown, which creates a mutual proximity between the film electrode layer and the elastomer electrode layer, but still no contact. In the next stage (**Fig. S6-III**), the sinusoidal actuation occurs and then reaches its peak. The film electrode layer and the elastomer electrode layer are squeezed against each other by the electric field force and the touch pressure and reach the maximum displacement. **Fig. S6-IV** shows that the vibrational. Displacement starts to bounce back due to reduced external voltage and increased elastic recovery force, and the actuator’s displacement reaches a reverse maximum state. As the sinusoidal voltage is varied, the actuator can produce constant motion in the vertical direction, thus providing haptic feedback forces.

**Note S2.** Actuator Theoretical Model Analysis.

To better understand the motion principles and elasticity of the flexible electrostatic actuator, we develop a corresponding spring-damped system model to explain the actuator's motion behavior. When considering the actuator as a whole, the forces acting on it include the pre-pressure exerted by the human hand, the electrostatic force generated by the alternating current (AC) applied between the electrode plates, and the final feedback force perceived by the hand, which results from the elastic restoring force of the upper film and the elasticity of the lower elastomer.


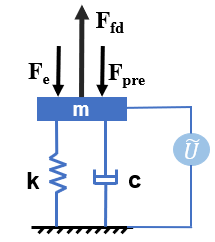


**Fig. S7.** The corresponding physical model of the actuator.

During actuator operation, the electrostatic force primarily comes from two components: the driving voltage V_𝑝𝑝_ applied to the electrode layers, and the energy storage V_0_ within the charged electret film. The driving voltage U is defined as a combination of constant bias voltage and AC voltage, with $F_{e}$​ representing the electrostatic force between the parallel plates. The driving voltage U is expressed as:

| U = V_0_ + V_𝑝𝑝_sin(𝜔t)/2 | (S1) |
| --- | --- |

V_0_ is the constant bias voltage, V_𝑝𝑝_ is the peak-to-peak driving voltage and 𝜔 is the angular frequency.

The electrostatic force between the two electrodes of the parallel plate capacitor structure is calculated using the Parallel plate capacitor equation:

| F_e_=$\frac{1}{2}\times\frac{\varepsilon_{0}\varepsilon s}{d^{2}}\times U^{2}$ | (S2) |
| --- | --- |

Here $\varepsilon_{0}$ is the absolute dielectric constant, 𝜀 is the relative dielectric constant, s is the area of the plates, d is the distance between the two plates and 𝑈 is the driving voltage.

| $U^{2}$=V_𝑝𝑝_^2^/4$\times$(1-$\cos(2\omega t)$)/2 +$V_{0}\times$V_𝑝𝑝_$\times\sin(\omega t)$ + $V_{0}^{2}$ | (S3) |
| --- | --- |

Substituting equation (S3), the electrostatic force is expressed as:

| F_e_=1/2$\times$𝜀_0_𝜀s/d^2^$\times$($V_{pp}^{2}$/8$- V_{pp}^{2}$cos(2𝜔t)/8 +$V_{0}V_{pp}\sin(\omega t)$+$V_{0}^{2}$) | (S4) |
| --- | --- |

Here, $V_{0}^{2}$ and $V_{pp}^{2}$ are constant terms, thus the electrostatic force is mainly affected by the middle two terms, and in the actual working process, when the driving voltage is extremely small, F_e_ is considered to be mainly determined by the V_0_𝑉_𝑝𝑝_sin(𝜔𝑡) term.

Therefore, the equation is reduced to constant and variable terms:

| $F_{e}$≈ C+1/2$\times$𝜀_0_𝜀_s_/d^2^$\times$V_0_V_𝑝𝑝_$\sin(\omega t)$= C + $F\sin(\omega t)$ | (S5) |
| --- | --- |

During the compression and recovery of the elastomer layer, the second-order spring damping system explains this motion process by describing the mechanical vibration motion of the haptic part as an equation:

| $F\sin\left( \omega_{d}t \right)=m\ddot{x}+c\dot{x}+kx$ | (S6) |
| --- | --- |

Here m is the overall mass of the actuator, k is the elasticity coefficient of the elastic layer of the actuator, c is the damping of the actuator material in motion, and F is the force applied to the actuator. 𝜔_0_=$\sqrt{k/m}$ represents the resonance frequency of the system, and the effect of the actuator's motion is maximized when the actuator is in the state of resonance when the amplitude of the vibration is shown in the following equation:

| $X_{max}=\frac{F}{2c}\times\sqrt{m/k}=F/2c\omega_{0}$ | (S7) |
| --- | --- |

X represents the displacement of the actuator surface and overall vibration amplitude. The motion of the thin-film electrode layer and elastomer electrode layer is considered as a unified system, and the corresponding spring-damping system's displacement under force is analyzed. Given that the thin-film electrode layer has limited potential for structural optimization, and its stiffness and thickness are constrained by the material properties, the elastic restoring force it can provide is relatively restricted. Therefore, refined theoretical modeling of the elastomer electrode layer is conducted to enhance its compressive deformation under electrostatic force and increase the elastic restoring force during electrostatic relaxation.

To address this, a multilayer PDMS elastomer model is proposed, improving upon the original single-stiffness PDMS by incorporating multiple stiffness layers. Each layer of the bilayer elastomer is modeled as a second-order spring-damped system, where $x_{1}，x_{2}$ represent the displacement of the elastomer under force in each layer; $k_{1}，k_{2}$ represent the elastic coefficients of the elastomer in each layer; and $m_{1}，m_{2}$ represent the mass of the elastomer in each layer; $c_{1}，c_{2}$ represent the damping of each layer of elastomer; $F_{1}\sin(\omega t), F_{2}\sin(\omega t)$ as the force term of each layer of elastomer.


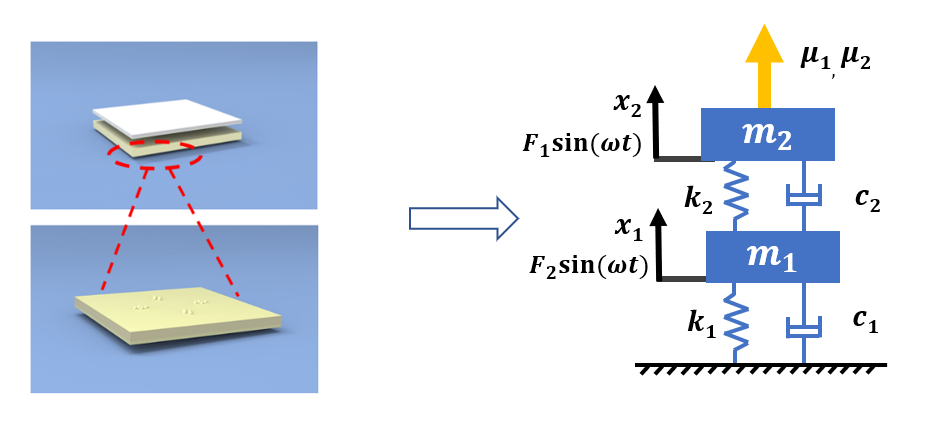


**Fig. S8.** The corresponding physical model of multilayer elastomer.

For the improved equivalent series second order spring damped system, the equations of motion for its mechanical vibration is written from Newton's second law:

| $m_{1}\ddot{x}_{1}=k_{2}{(x}_{2}-x_{1})-k_{1}x_{1}-c_{1}\dot{x}_{1}+F_{1}\sin(\omega t)$  $m_{2}\ddot{x}_{2}=- k_{2}{(x}_{2}-x_{1})-c_{2}\dot{x}_{2}+F_{2}\sin(\omega t)$ | (S8) |
| --- | --- |

To simplify the equation, a =$\frac{k_{1}+k_{2}}{m_{1}}$, b =$\frac{k_{2}}{m_{1}}$, c =$\frac{k_{2}}{m_{2}}$, $p_{1}$=$\frac{F_{1}}{m_{1}}$, $p_{2}$=$\frac{F_{2}}{m_{2}}$，$q_{1}$=$\frac{c_{1}}{m_{1}}$, $q_{2}$=$\frac{c_{2}}{m_{2}}$, then,

| $\ddot{x}_{1}$+$q_{1}\dot{x}_{1}+ax_{1}-bx_{2}$=$p_{1}\sin(\omega t)$  $\ddot{x}_{2}+{q_{2}\dot{x}}_{1}-cx_{1}$+$cx_{2}$=$p_{2}\sin(\omega t)$ | (S9) |
| --- | --- |

The solutions of the above equation can be categorized into 1) the homogeneous solution representing the free vibration of the spring-damped system, and 2) the non-homogeneous particular solution representing the forced vibration of the actuator under force. During the operation of the actual actuator, the damping causes the free vibration to decay to zero very quickly. This work focuses on solving the non-homogeneous particular solution of the system's equations to develop a realistic vibration model for the actuator.

Let the particular solution of the system of equations be:

| $x_{1}$=$B_{1}\sin(\omega t)$  $x_{2}$=$B_{2}\sin(\omega t)$ | (S10) |
| --- | --- |

$x_{1}, x_{2}$ represent the vibration displacements of the double-layer elastomer at a frequency equal to the frequency of the AC signal applied to the actuator, and $B_{1}$, $B_{2}$ are the vibration amplitudes of the double-layer elastomer. Substituting the particular solution into the system of equations is obtained:

| (a-$\omega^{2}$)$B_{1}-$b$B_{2}$=$p_{1}$  -c$B_{1}$+ (c-$\omega^{2}) B_{2}$=$p_{2}$ | (S11) |
| --- | --- |

To obtain a particular solution to the system of equations implies that a non-zero solution to $B_{1}$, $B_{2}$ is required, then the determinant of the coefficients of the equation is zero:

| Δ = $\left\vert\begin{matrix} a-\omega^{2} & -b \\ -c & c-\omega^{2} \end{matrix} \right\vert$ = (a−ω^2^) (c−ω^2^) − bc | (S12) |
| --- | --- |

Let Δ = 0, the two intrinsic frequencies $\omega_{n1}$ (the first intrinsic frequency of the system), $\omega_{n2}$ (the second intrinsic frequency of the system) of the double-layer elastomer is solved for, and $\omega_{n1}<\omega_{n2}$, and the two intrinsic frequencies is specifically written as:

| ω_𝑛1_ = $\frac{a+c}{2}-\sqrt{{(\frac{a-c}{2})}^{2}+\mathrm{bc}}$  ω_𝑛2_ = $\frac{a+c}{2}+\sqrt{{(\frac{a-c}{2})}^{2}+\mathrm{bc}}$ | (S13) |
| --- | --- |

According to Clem's Law, the solution is:

| $\Delta_{1}$=$\left\vert\begin{matrix} p_{1} & -b \\ p_{2} & c-\omega^{2} \end{matrix} \right\vert$ | (S14) |
| --- | --- |
| $\Delta_{2}$=$\left\vert\begin{matrix} a-\omega^{2} & p_{1} \\ -c & p_{2} \end{matrix} \right\vert$ | (S15) |
| $B_{1}$=$\frac{\Delta_{1}}{\Delta}$=$\frac{\left( k_{2}-m_{2}\omega^{2} \right)F_{1}+k_{2}F_{2}}{\left( k_{1}+k_{2}-m_{1}\omega^{2} \right)\left( k_{2}-m_{2}\omega^{2} \right)-k_{2}^{2}}$ | (S16) |
| $B_{2}$=$\frac{\Delta_{2}}{\Delta}$=$\frac{\left( {F_{1}+k}_{2}-m_{1}\omega^{2} \right)F_{2}+k_{2}F_{1}}{\left( k_{1}+k_{2}-m_{1}\omega^{2} \right)\left( k_{2}-m_{2}\omega^{2} \right)-k_{2}^{2}}$ | (S17) |

The above equations demonstrate that the mechanical vibration of the elastomer in the actuator is primarily influenced by the driving electrical signal. Specifically, the vibration frequency matches that of the driving signal, while the vibration amplitude is affected by both the amplitude and frequency of the signal, as well as the material properties and elastic behavior of the actuator.

When the driving signal's frequency approaches the system's natural frequency, the vibration amplitude increases significantly. At resonance, when the driving frequency equals the system's natural frequency, defined by (a－$\omega^{2}$)(c－$\omega^{2}$)－bc = 0, the amplitude reaches its maximum, a phenomenon known as resonance. Although theoretically, resonance leads to infinite amplitude, damping effects in reality limit the amplitude to a finite value. However, in the equivalent model analysis, the general behavior of vibration and methods to enhance amplitude remain unaffected by the damping term, which is thus neglected in subsequent amplitude studies. The vibration characteristics of the multilayer elastomer actuator are examined when the frequency is near the natural frequency.

In a double-degree-of-freedom spring-damped system, two resonant frequencies, $\omega_{n1}$ and $\omega_{n2}$, each associated with a specific amplitude ratio. Under the stimulation of a fixed driving electric signal, we can obtain a definite displacement ratio and a definite amplitude ratio at any instant.

Thus, its amplitude ratio is listed as:

| $\left\vert\frac{B_{2}}{B_{1}} \right\vert_{\omega_{n1}}$=$\frac{\left( {F_{1}+k}_{2}-m_{1}{\omega_{n2}}^{2} \right)F_{2}+k_{2}F_{1}}{\left( k_{2}-m_{2}{\omega_{n2}}^{2} \right)F_{1}+k_{2}F_{2}}$=$\mu_{1}$ | (S18) |
| --- | --- |
| $\left\vert\frac{B_{2}}{B_{1}} \right\vert_{\omega_{n2}}$=$\frac{\left( {F_{1}+k}_{2}-m_{1}{\omega_{n2}}^{2} \right)F_{2}+k_{2}F_{1}}{\left( k_{2}-m_{2}{\omega_{n2}}^{2} \right)F_{1}+k_{2}F_{2}}$=$\mu_{2}$ | (S19) |

The amplitude ratio can represent the vibration pattern of a system. When the system vibrates according to the intrinsic frequency, the amplitude ratio depends only on the physical properties of the system itself and is independent of the initial conditions of the motion.

In this equation, $\mu_{1}$ represents a first principal vibration pattern, corresponding to the amplitude ratio of the first intrinsic frequency $\omega_{n1}$, and $\mu_{2}$ represents a second principal vibration pattern, corresponding to the amplitude ratio of the second intrinsic frequency $\omega_{n2}$. When the system is driven according to any one of the intrinsic frequencies, the vibration at that frequency is referred to as the main vibration pattern of the system.

Setting $F_{1}$= 0 simplifies the above equation and it is obtained:

| $\mu_{1}$=$\left\vert\frac{k_{2}-m_{1}{\omega_{n1}}^{2}}{k_{2}} \right\vert$ | (S20) |
| --- | --- |
| $\mu_{2}$=$\left\vert\frac{k_{2}-m_{1}{\omega_{n2}}^{2}}{k_{2}} \right\vert$ | (S21) |

In order to maximize the two-order principal vibration mode, substitute ${\omega_{n1}, \omega}_{n2}$in equation (13), the ratio of the stiffness of two-layer elastomer $k_{1}=\tau k_{2}$ and the ratio of the mass $m_{1}=\sigma m_{2}$, and then the actuator principal vibration mode function with elastomer stiffness as well as the mass parameter is obtained finally.

| $\mu_{1}$= 1 – $\sigma\frac{k_{2}}{m_{2}}{( \frac{1+\tau+\sigma}{2\sigma}-\sqrt{\frac{\tau^{2} +\sigma^{2} +2\tau+2\sigma-2\tau\sigma+1}{4\sigma^{2}}})}^{2}$ | (S22) |
| --- | --- |
| $\mu_{2}$= 1 – $\sigma\frac{k_{2}}{m_{2}}{( \frac{1+\tau+\sigma}{2\sigma}+\sqrt{\frac{\tau^{2} +\sigma^{2} +2\tau+2\sigma-2\tau\sigma+1}{4\sigma^{2}}})}^{2}$ | (S23) |

**Table S1.** Measured Young’s modulus and Poisson’s ratio for elastomers with different cross-linking ratios used in this study. The measurements are performed using the Instron ElectroPuls Dynamic Test System (E3000, Instron) and a Universal Spring Force Tension and Compression Tester (WDT-10).

| Cross-linking ratio | Young’s modulus (MPa) | Poisson’s ratio |
| --- | --- | --- |
| 5:1 | 1.971 | 0.396 |
| 10:1 | 1.611 | 0.402 |
| 15:1 | 0.745 | 0.397 |
| 20:1 | 0.470 | 0.405 |
| 25:1 | 0.256 | 0.399 |

**Table S2.** The designed stiffness schemes for the multilayer elastomers.

| **Solution: Cross-linking ratio** | | **First layer** | **Second layer** | **Third layer** |
| --- | --- | --- | --- | --- |
| Single  layer | Scenario 1 | 5:1 | \ | \ |
|  | Scenario 2 | 10:1 | \ | \ |
|  | Scenario 3 | 20:1 | \ | \ |
|  | Scenario 4 | 25:1 | \ | \ |
| Double  layer | Scenario 5 | 10:1 | 20:1 | \ |
|  | Scenario 6 | 20:1 | 10:1 | \ |
| Triple  layer | Scenario 7 | 20:1 | 15:1 | 10:1 |
|  | Scenario 8 | 25:1 | 15:1 | 10:1 |
|  | Scenario 9 | 20:1 | 15:1 | 5:1 |
|  | Scenario 10 | 25:1 | 15:1 | 5:1 |


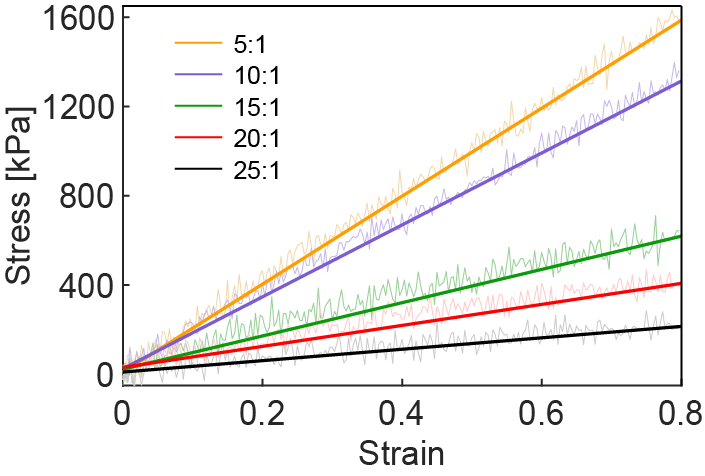


**Fig. S9.** Measured Young’s modulus of the elastomers with different cross-linking ratios.


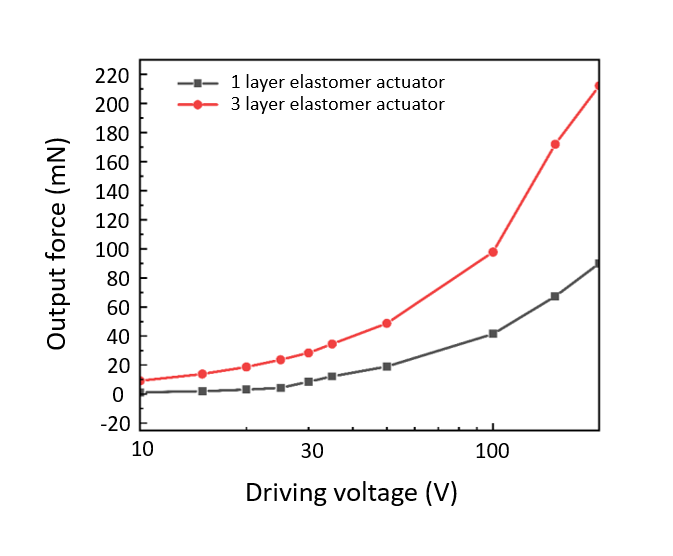


**Figure S10.** Measured the output force of single-layer and multilayer elastomer actuators under an applied driving voltage from 10 V to 200 V.


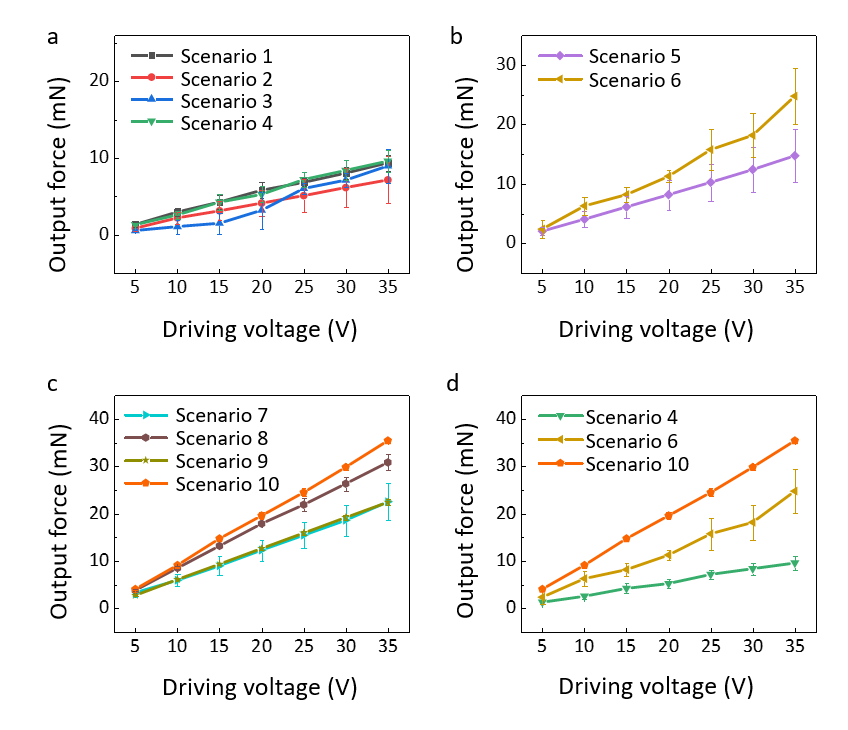


**Fig. S11.** Measured the output force of multilayer elastomer actuators with ten stiffness scenarios under an applied driving voltage from 5 to 35 V.

**a**, Measured output force-driving voltage of single-layer PDMS. **b**, Measured output force-driving voltage of double-layer PDMS. **c**, Measured output force-driving voltage of triple-layer PDMS. **d**, Comparing the best single-, double- and triple-layer elastomer actuator output forces.

**Note S3.** Electric field superposition of elastomer actuators by electret films.


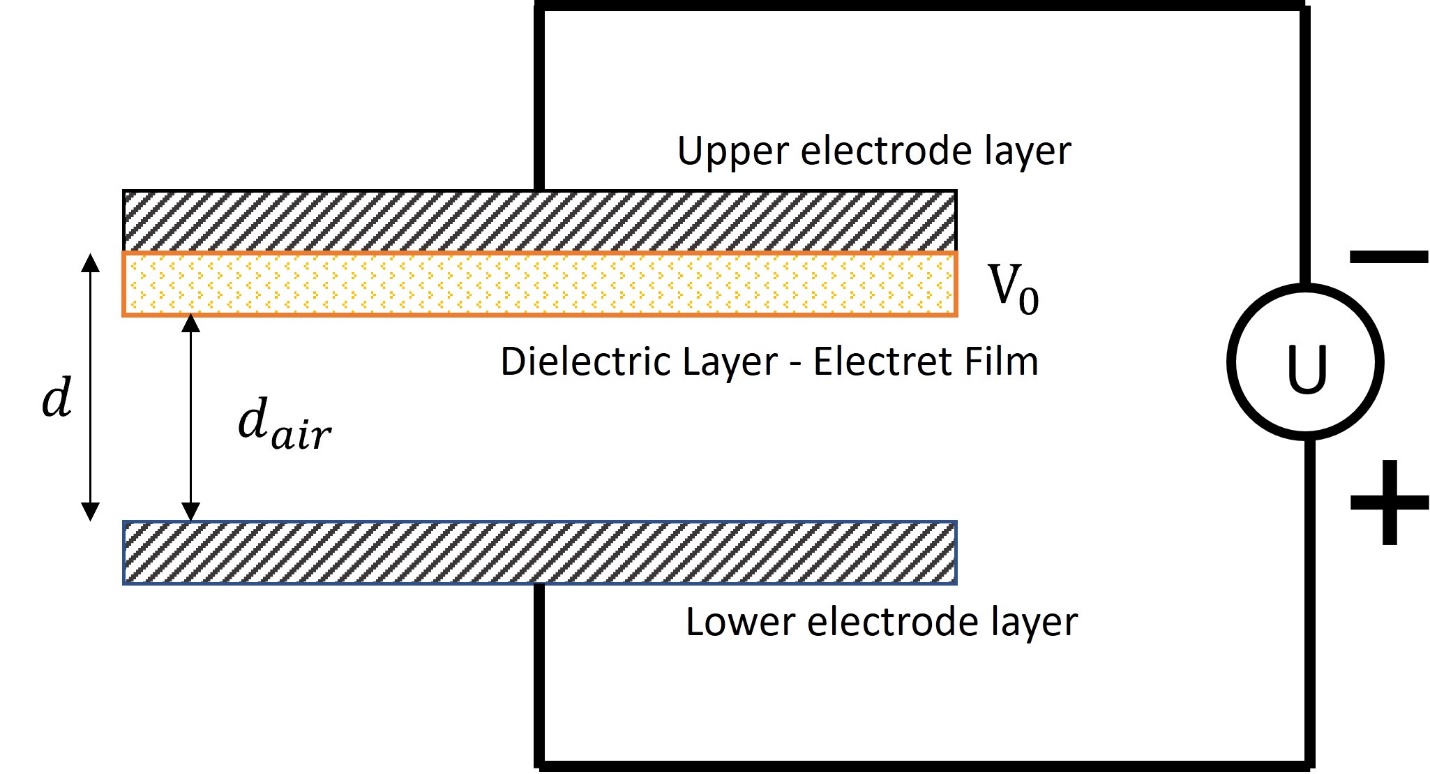


**Fig. S12.** Equivalent electrical modeling of electret actuator.

The electric field models of the electret actuator without and with the charged FEP electret material as a dielectric film are established in **Fig. S10 and 11**.


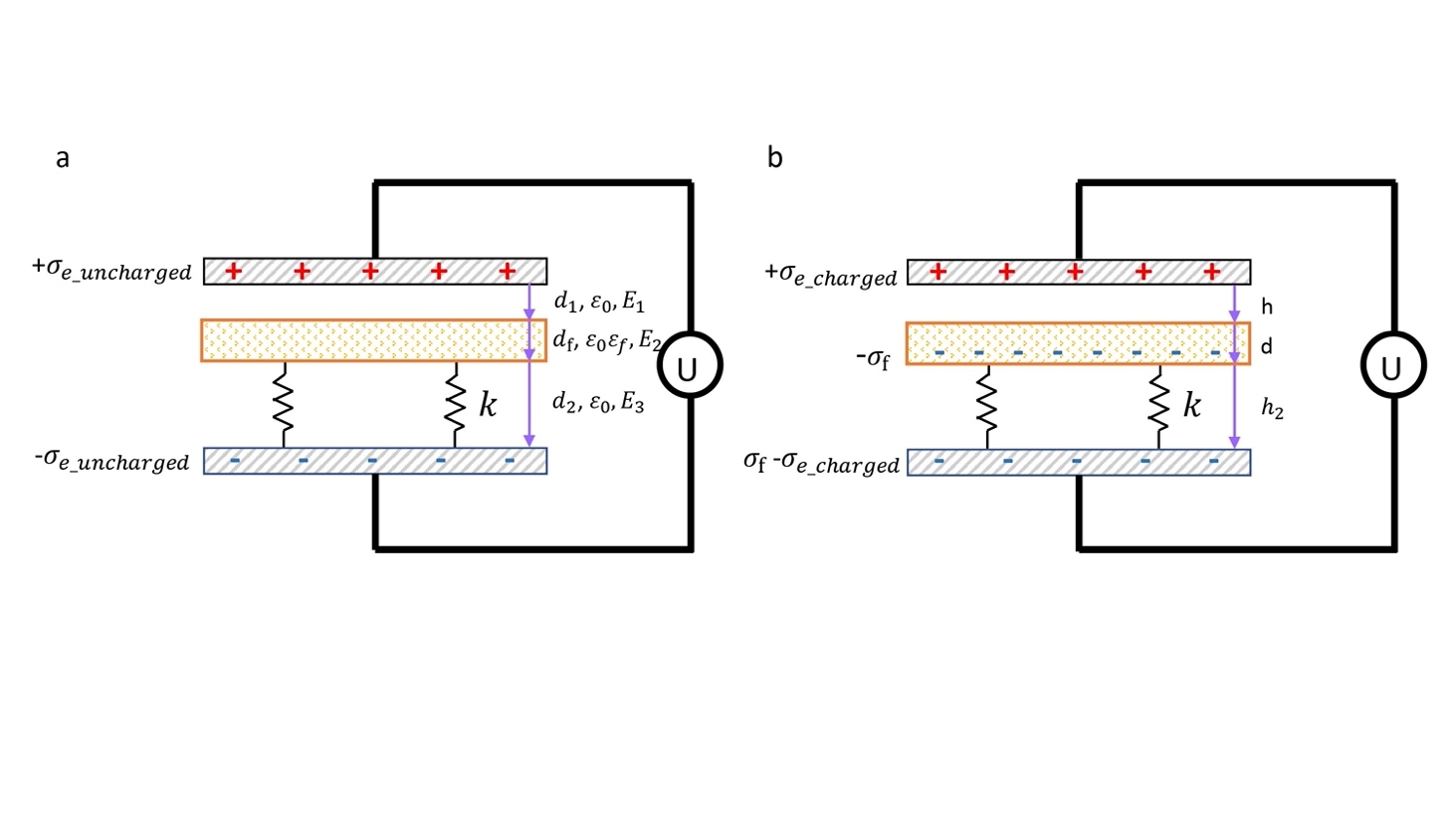


**Fig. S13.** Electric field models of the actuator with (A) uncharged and (B) charged electret films.

According to Gauss's law, the relationship between the electric field strength and the surface charge density is established:

| $\varepsilon_{0}E_{1}=\sigma_{e}$ | (S24) |
| --- | --- |
| $\varepsilon_{0}\varepsilon_{f}E_{2}=\sigma_{e}$ | (S25) |
| $\varepsilon_{0}E_{3}=\sigma_{e}$ | (S26) |

The potential difference between the upper and lower electrode layers is written as:

| U(t) = $E_{1}d_{1}\left( t \right)+E_{2}d_{f}\left( t \right)+E_{3}d_{2}\left( t \right)$  = $\sigma_{e}\left( \frac{d_{1}\left( t \right)}{\varepsilon_{0}}+\frac{d_{f}\left( t \right)}{\varepsilon_{0}\varepsilon_{f}}+\frac{d_{2}\left( t \right)}{\varepsilon_{0}} \right)$ | ((S27) |
| --- | --- |

For the model without the corona charging process, the electrostatic actuator is driven by the electric field force induced by the voltage applied to the upper and lower electrode layers. Thus, the relationship between the charges on the electrodes and the applied voltage is described as:

| U =$\sigma_{e\_uncharged}\left( \frac{d_{1}}{\varepsilon_{0}}+\frac{d_{f}}{\varepsilon_{0}\varepsilon_{f}}+\frac{d_{2}}{\varepsilon_{0}} \right)$ | ((S28) |
| --- | --- |

The equivalent capacitance is calculated as:

| $C_{\mathrm{uncharged}}=\frac{{A\sigma}_{e\_uncharged}}{U}= \frac{A\varepsilon_{0}}{d_{1}+\frac{d_{f}}{\varepsilon_{f}}+d_{2}}$ | ((S29) |
| --- | --- |

The energy of the electrostatic field for parallel plate capacitors is:

| $W_{\mathrm{uncharged}}=\frac{1}{2}C_{\mathrm{uncharged}}U^{2}=\frac{1}{2} \frac{A\varepsilon_{0}}{d_{1}+\frac{d_{f}}{\varepsilon_{f}}+d_{2}}U^{2}$ | ((S30) |
| --- | --- |

The Maxwell stress on the upper electrode is expressed as:

| $P_{\mathrm{uncharged}}=\frac{1}{A}\frac{\partial W_{\mathrm{uncharged}}}{\partial{(d}_{2})}=-\frac{1}{2} \frac{\varepsilon_{0}}{(d_{1}+\frac{d_{f}}{\varepsilon_{f}}+d_{2})^{2}}U^{2}$ | ((S31) |
| --- | --- |

With the use of a charged electret material as a dielectric film, the relationship between the charges on the electrodes and the applied voltage is described as:

| U =$\sigma_{e\_charged}\left( \frac{d_{1}}{\varepsilon_{0}}+\frac{d_{f}}{\varepsilon_{0}\varepsilon_{f}}+\frac{d_{2}}{\varepsilon_{0}} \right)-\sigma_{f}\frac{d_{2}}{\varepsilon_{0}}$ | ((S32) |
| --- | --- |

Similarly, the equivalent capacitance is calculated as:

| $C_{\mathrm{charged}}=\frac{{A\sigma}_{e\_charged}}{U}= \frac{A\varepsilon_{0}}{d_{1}+\frac{d_{f}}{\varepsilon_{f}}+d_{2}}+\frac{\sigma_{f}d_{2}A}{U(d_{1}+\frac{d_{f}}{\varepsilon_{f}}+d_{2})}$ | ((S33) |
| --- | --- |

The energy of the electrostatic field for parallel plate capacitors is:

| $W_{\mathrm{charged}}=\frac{1}{2}C_{\mathrm{charged}}U^{2}=\frac{1}{2} \frac{A\varepsilon_{0}}{d_{1}+\frac{d_{f}}{\varepsilon_{f}}+d_{2}}U^{2}+\frac{1}{2}\frac{\sigma_{f}d_{2}AU}{d_{1}+\frac{d_{f}}{\varepsilon_{f}}+d_{2}}$ | ((S34) |
| --- | --- |

The driving stress on the upper electrode is calculated as:

| $P_{\mathrm{charged}}=\frac{1}{A}\frac{\partial W_{\mathrm{charged}}}{\partial(d_{2})}=-\frac{1}{2} \frac{\varepsilon_{0}}{(d_{1}+\frac{d_{f}}{\varepsilon_{f}}+d_{2})^{2}}U^{2}-\frac{1}{2}\frac{\sigma_{f}d_{2}U}{(d_{1}+\frac{d_{f}}{\varepsilon_{f}}+d_{2})^{2}}$ | ((S35) |
| --- | --- |

It is clear that the charged electret film brings a larger effective capacitance and higher electrostatic field energy to the electrical model. The Maxwell stresses of the electrical model using the charged electret film gain the additional stresses introduced by the electret. Thus, the charged electret material can replace the DC bias. Compared to the uncharged model, a several-fold increase in output force is realized after charging.


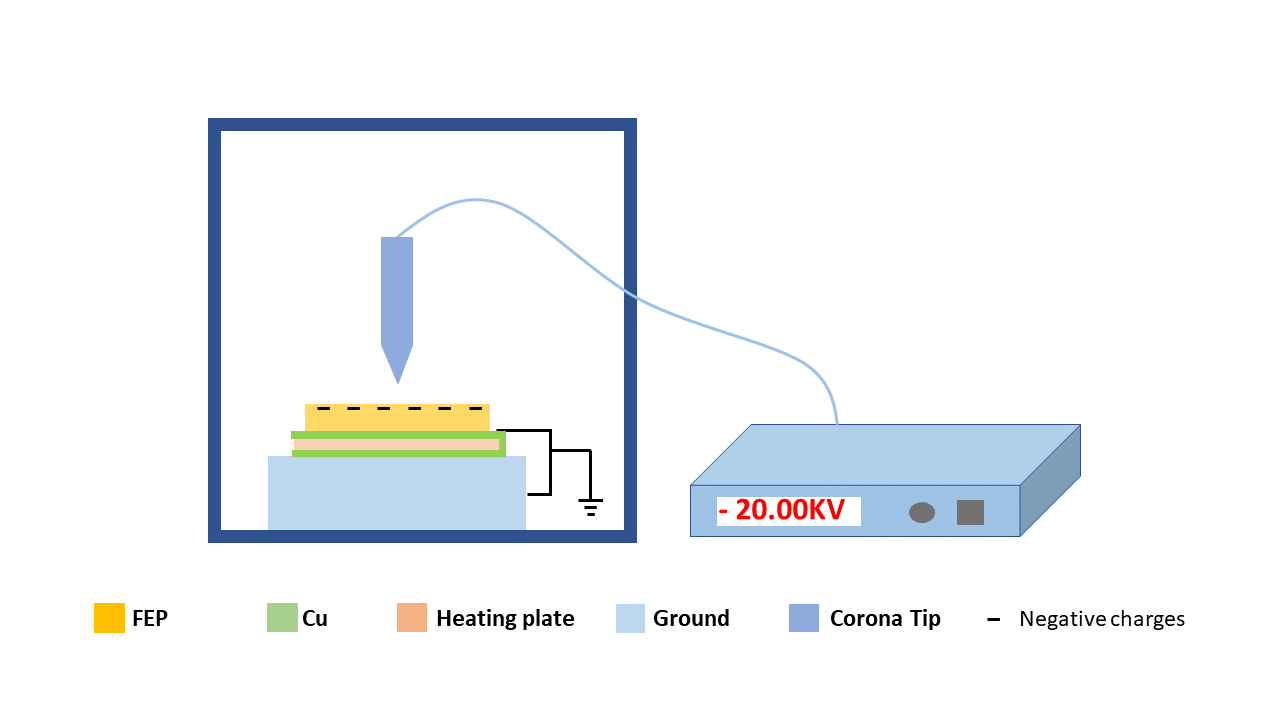


**Fig. S14.** Schematic diagram of the high-temperature corona charging for the FEP electret film.


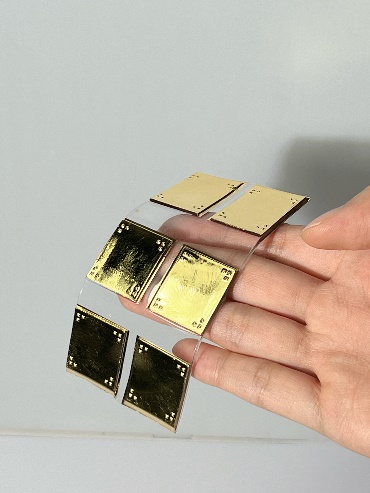

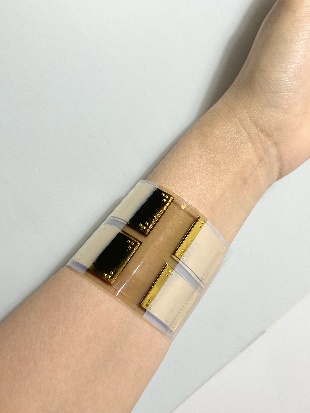

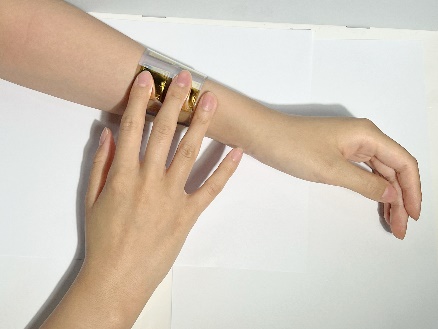


**Fig. S15.** Optical image of arrayed flexible haptic interface and interaction morphology with human arm.


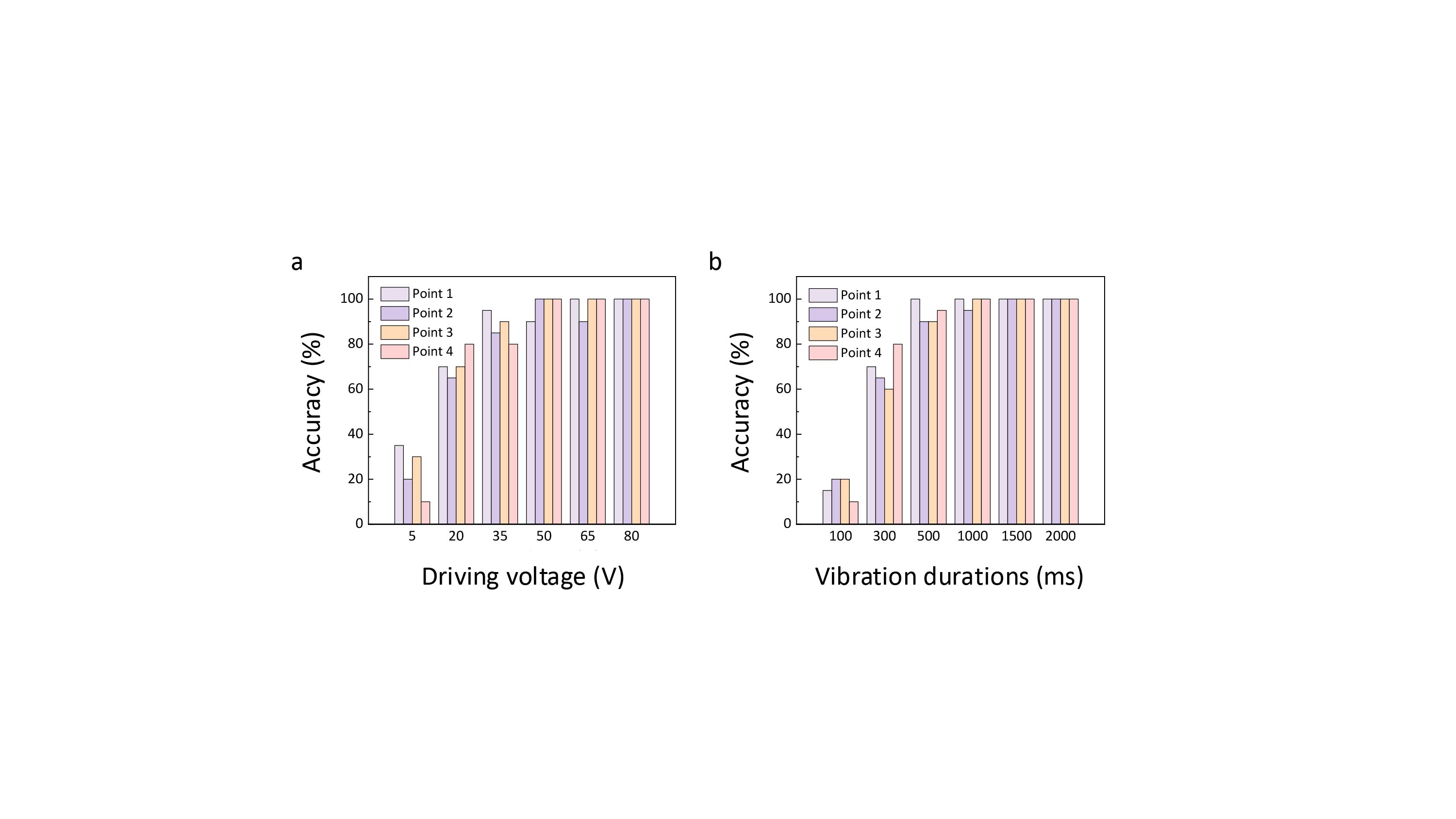


**Fig. S16.** Manual discrimination of actuator outputs.

In order to test the tactile discriminationability of the arrayed actuators. The experimental design is driven by different a) voltages and b) vibration durations of four points that are played continuously. Volunteers touch the haptic interface and identify the four vibrating points and their vibration order. The experiment demonstrates that the haptic interface can generate discernible output feedback forces in terms of vibration intensity, vibration order, and vibration location.

**Table S3.** Comparison of performance and material design between previously reported flexible actuators and the actuator developed in this work.

| Principle | Work | Driving  Voltage | Output  force | Electrode material design | Elastomer stiffness control |
| --- | --- | --- | --- | --- | --- |
| Electrostatic | This  work | 35 V | 34.4 mN | Film + multilayer PDMS elastomer | Yes |
|  |  | 200 V | 212.3 mN |  |  |
|  | [26] | 200 V | 90 mN | Film + PDMS elastomer | No |
|  | [21] | 500 V | 20 mN | Film | / |
|  | [40] | 1400 V | 300 mN | Film | / |
|  | [52] | 1500 V | 1200 mN | PVC Gel + FR4 | / |
|  | [42] | 4000 V | 2300 mN | Film | / |
|  | [39] | 100 V | 20 mN | Textile | / |
|  | [41] | 500 V | 80 mN | Film | / |
|  | [47] | ~ 275 V | 9 mN | PDMS elastomer | No |
|  | [49] | 1200 V | 250 mN | Film | / |
|  | [45] | 1000 V | 450 mN | PDMS Elastomer | No |
|  | [51] | 4000 V | 30 mN | PDMS Elastomer | No |
|  | [44] | 6000 V | 42 mN | PDMS Elastomer + AAm-NaCl hydrogel | No |
|  | [53] | 6000 V | 2300 mN | Film + Ecoflex elastomer | No |
|  | [50] | 8000 V | 80 mN | Film + FR4 | / |
| Electroactive | [48] | 4000 V | 255 mN | Film + PDMS elastomer | No |
|  | [46] | 10000 V | 200 mN | PDMS + ecoflex Elastomer | Yes |
|  | [43] | 2 V | 0.35 mN | Film | / |

**Fig. S17.** The effect of frequency change on haptic arousal.

This experiment is designed with six haptic stimuli of different frequencies driven under the same voltage. Users can distinguish between high and low-frequency stimuli. Among them, low-frequency haptic vibrations are more relaxing to the users, while high-frequency vibrations increase arousal. However, after 175 Hz, the arousal level increases, but the difference is not significant. This may be because the human hand is not very sensitive to "high frequency" and "higher frequency".


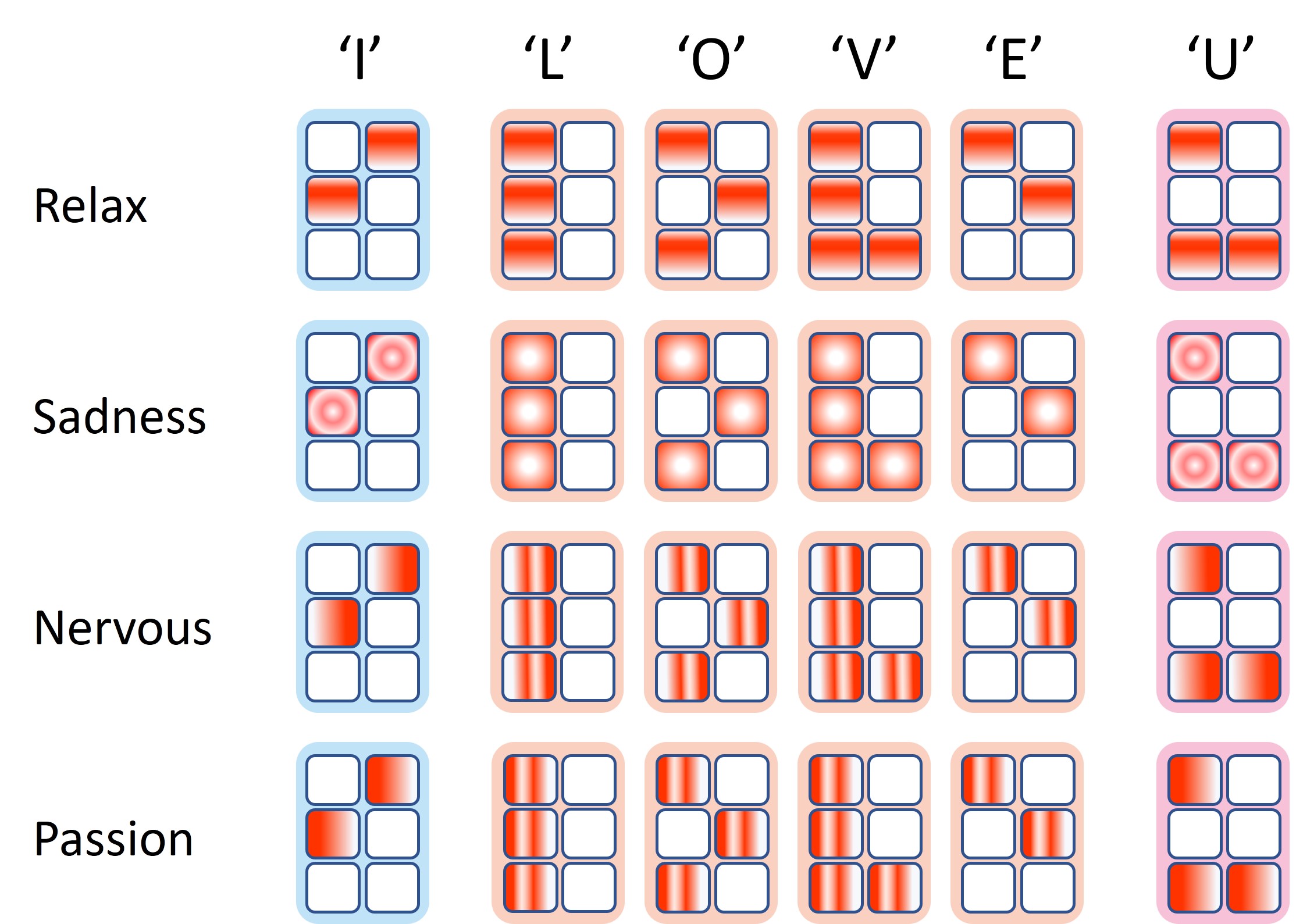


**Fig. S18.** Schematic illustration of emotionally driven electronic Braille vibrations for the phrase “I love U.”

The vibration points translate the text into Braille based on the international Braille correspondence table. Different touch sensations for four fundamental emotions can be simulated through the actuated programming of the haptic interface. The electronic Braille output times for the different emotions vary according to the expression of the emotion, just as the human tone of voice varies.

**Note S4.** Detailed design steps and experimental data for navigational haptic.

First, the effect of the actuation flow direction (AFD) between two vibration points on the real touch direction is experimentally determined; then, experiments on the driving amplitude (A) and the overlapping vibration time (OVT) of the driving force between the two vibration points are designed to obtain a haptic driving scheme for the optimal navigation between the two points. Finally, the haptic actuation for navigation between two points is extended to four and multiple points. The haptic interface for actuation flow direction (AFD), actuation onset time (AOT), overlap vibration time (OVT), and actuation amplitude (A) for navigation commands is determined.


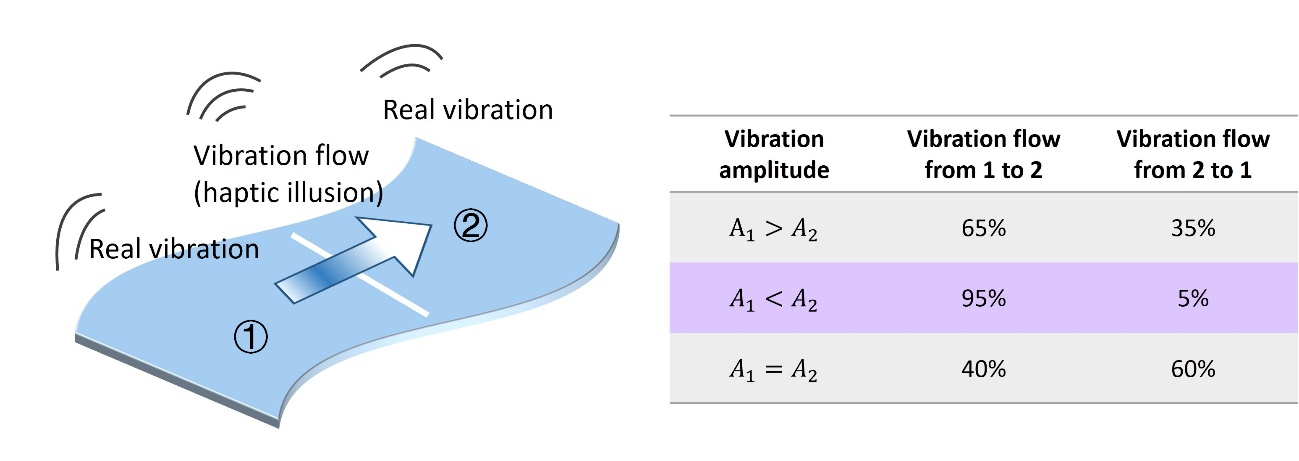


**Fig. S19.** Flow direction test for haptic illusions.

The results show that the direction of vibration flow is more acceptably perceived when the stimuli starts from a low amplitude vibration point to a high amplitude vibration point, which is better than the same amplitude as well as the amplitude being stronger and then weaker.


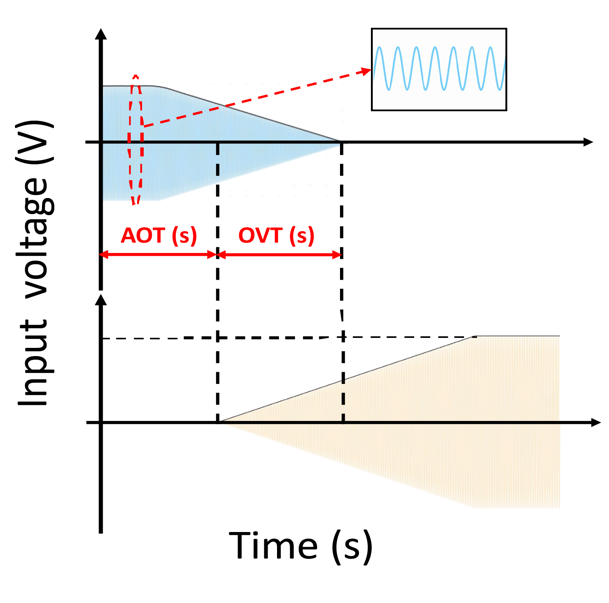


**Fig. S20.** Schematic design of the driving signal for haptic illusion between two points. The driving signal is a sinusoidal voltage stimulus. Two-point haptic illusion is realized by programming the two drive signals shown in **Table S4**.

**Table S4.** The design scheme of haptic illusion between two points.

|  | A_1_(V) | A_2_ (V) | $\Delta\boldsymbol{t}$(s) |
| --- | --- | --- | --- |
| Mode 1 | 15 | 25 | 0.2 |
| Mode 2 | 15 | 25 | 0.4 |
| Mode 3 | 15 | 25 | 0.6 |
| Mode 4 | 15 | 25 | 0.8 |


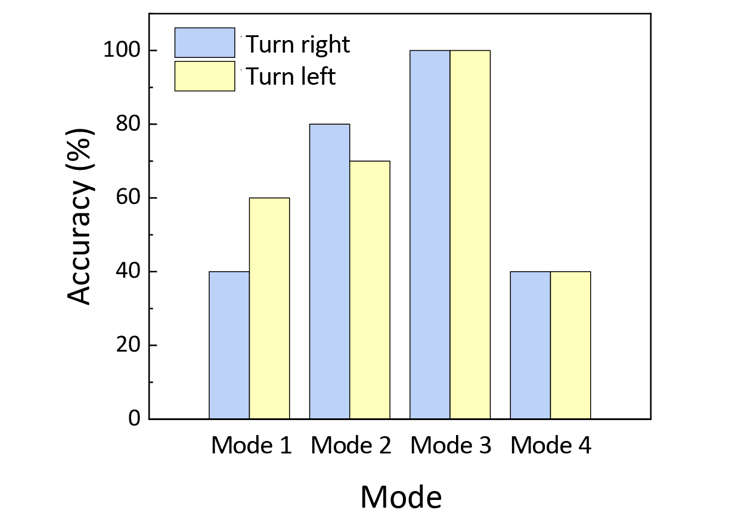


**Fig. S21.** Touch accuracy in the two-point haptic illusion model. Under the condition of fixed actuation amplitude (A), **Fig. S13** demonstrates that the optimal haptic illusion effect can be realized when the actuation onset time (AOT) is 0.6 s and the overlap vibration time (OVT) is 0.2 s. Therefore, based on the above results, the driving signal for the haptic illusion between two points is determined as shown in Mode 3 in **Table S4**.


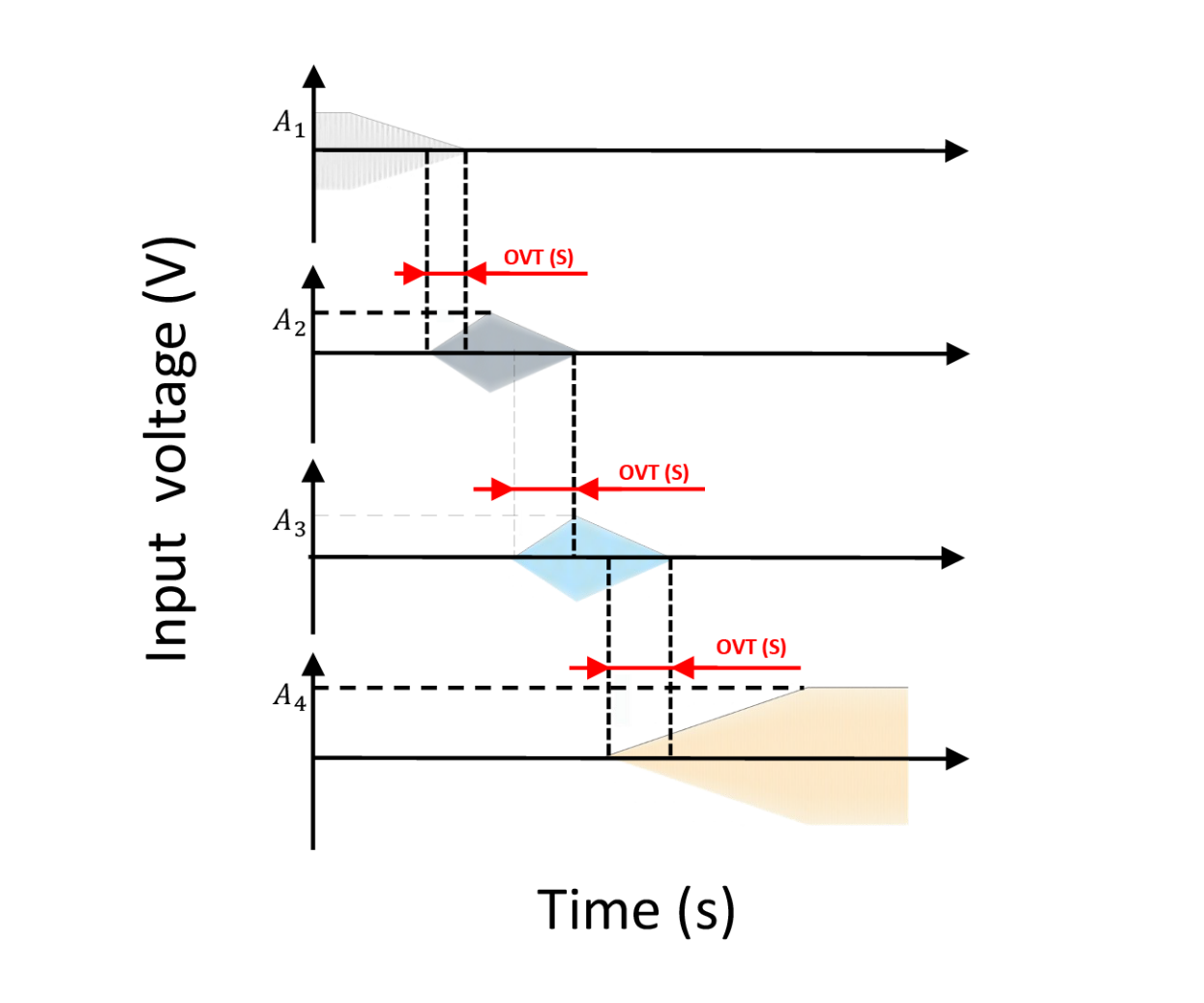


**Fig. S22.** Schematic design of the driving signal for haptic illusion between four points. The driving scheme between the four points follows the same OVT (200 ms) and actuation amplitude rules (from weak to strong) as in the previous experiment for the haptic illusion between two points. Four-point haptic illusion is realized by programming the four drive signals shown in **Table S5**.

**Table S5.** The design scheme of haptic illusion between four points

|  | **A_1_(V)** | **A_2_ (V)** | **A_3_ (V)** | **A_4_ (V)** | $\boldsymbol{\Delta}\boldsymbol{t}$**(s)** |
| --- | --- | --- | --- | --- | --- |
| **Mode 1**  $A_{1}=A_{2}=A_{3}=A_{4}$ | 20 | 20 | 20 | 25 | 0.2 |
| **Mode 2**  $A_{1}<A_{2}=A_{3}<A_{4}$ | 15 | 20 | 20 | 25 | 0.2 |
| **Mode 3**  $A_{1}>A_{2}=A_{3}<A_{4}$ | 25 | 20 | 20 | 25 | 0.2 |


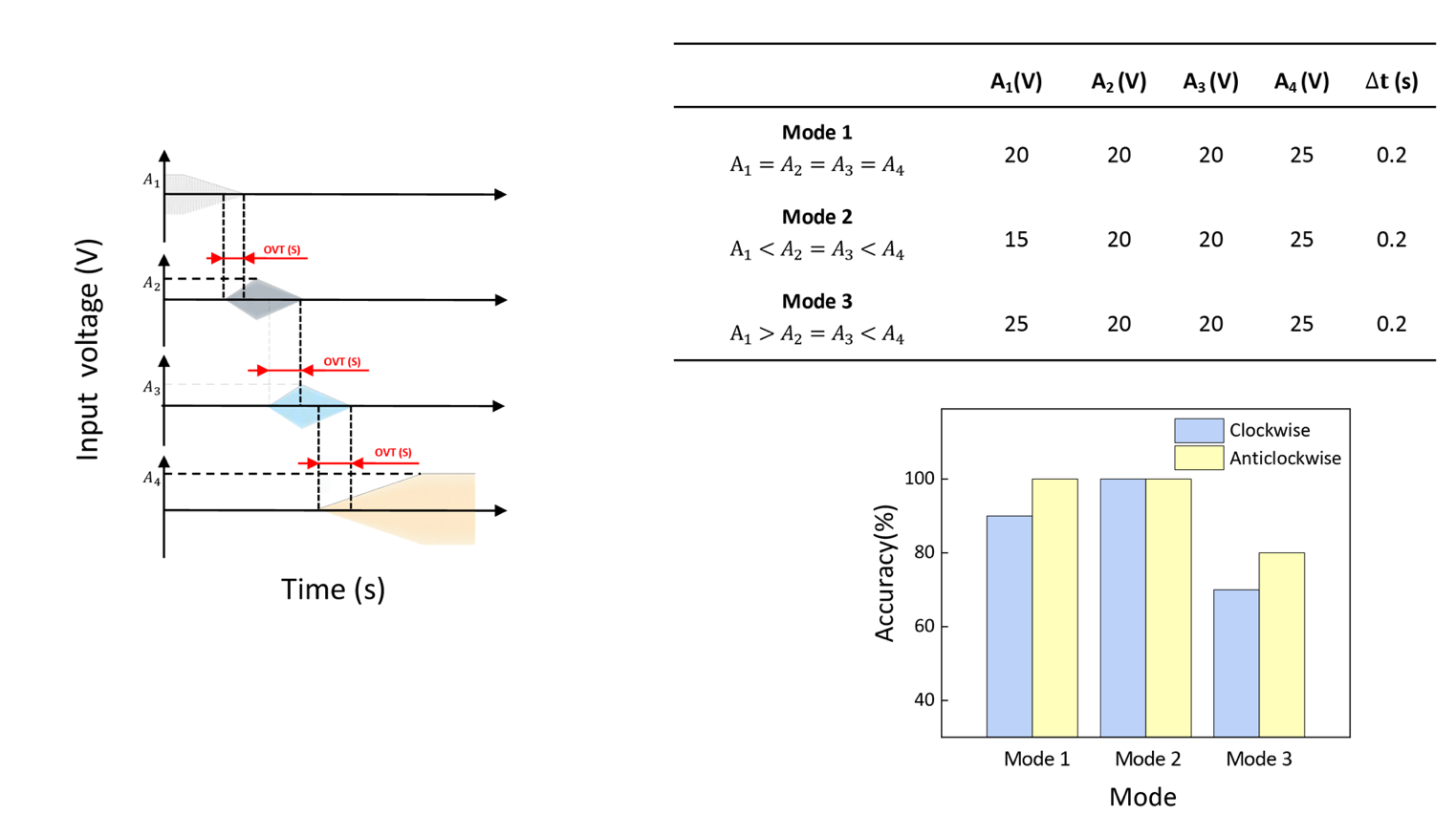


**Fig. S23.** Touch accuracy in the four-point haptic illusion model. The optimal vibration amplitude is determined as shown in Mode 2 in **Table S5**.

**Table S6.** Navigation commands with haptic flow sensation based on the haptic illusion.

|  |  | **Mode 1** | **Mode 2** |
| --- | --- | --- | --- |
| **Left/right** |  | 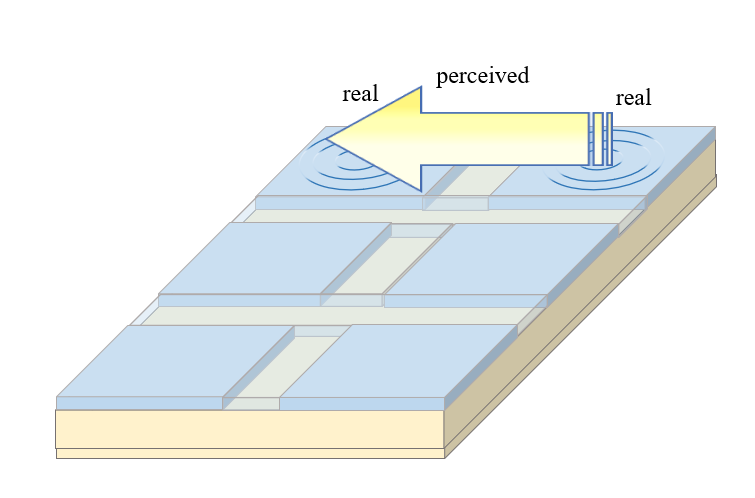 | 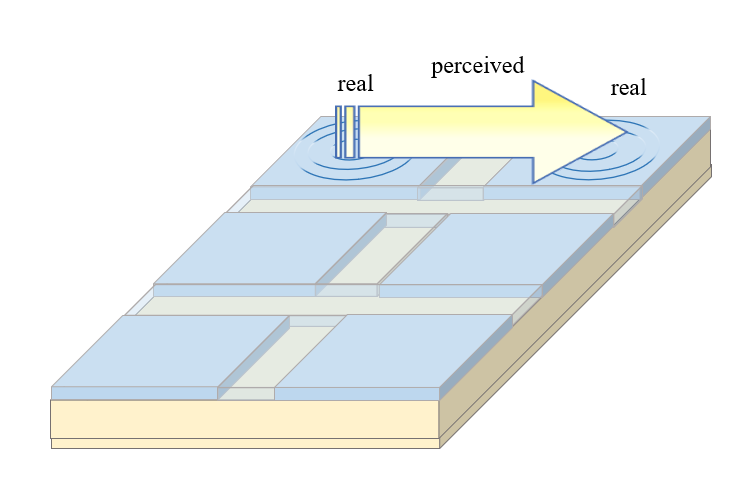 |
| **Oblique forward** |  | 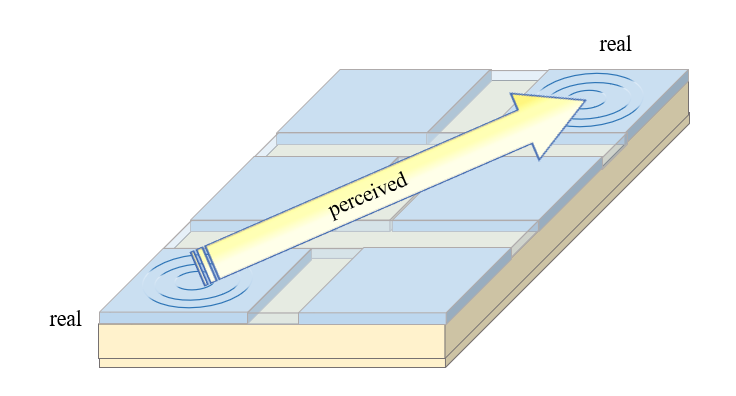 | 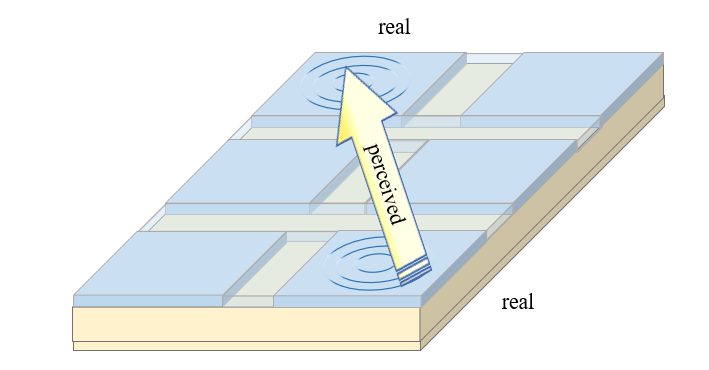 |
| **Clockwise** |  | 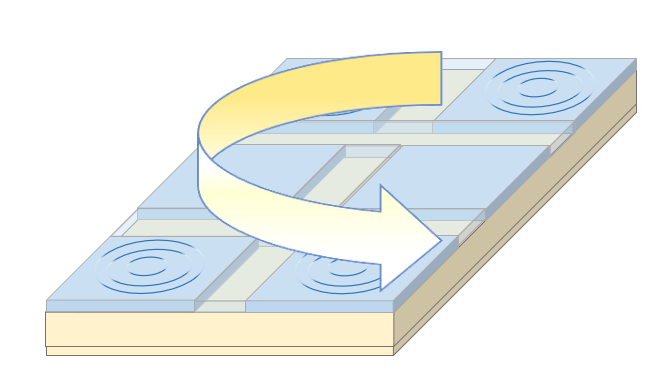 | 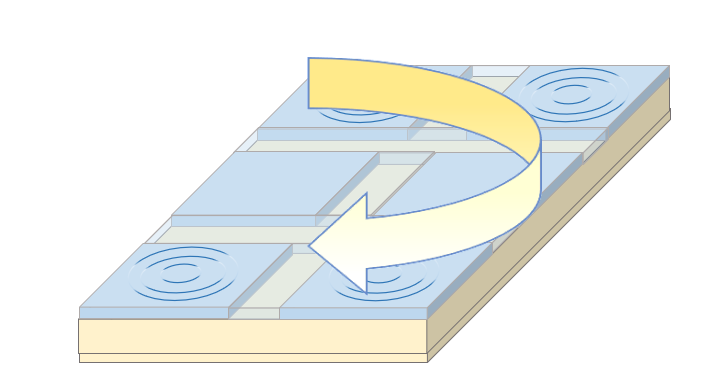 |
| **Front/back** |  | 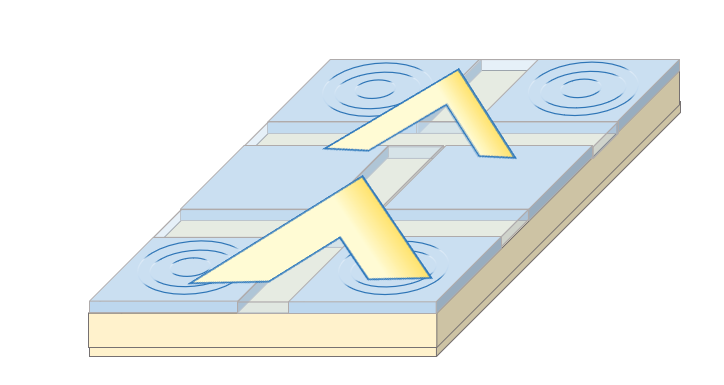 | 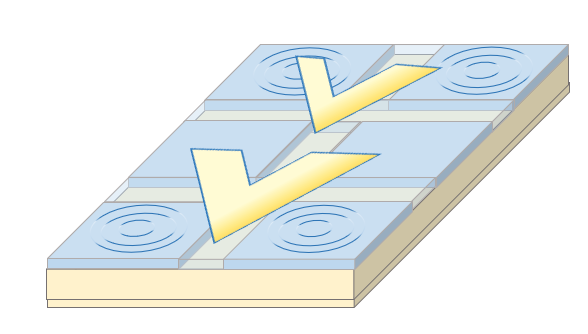 |
| **Arrival** |  | 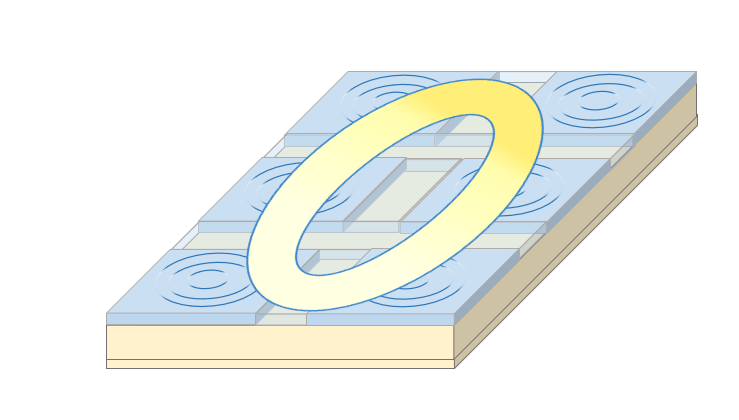 |  |

**
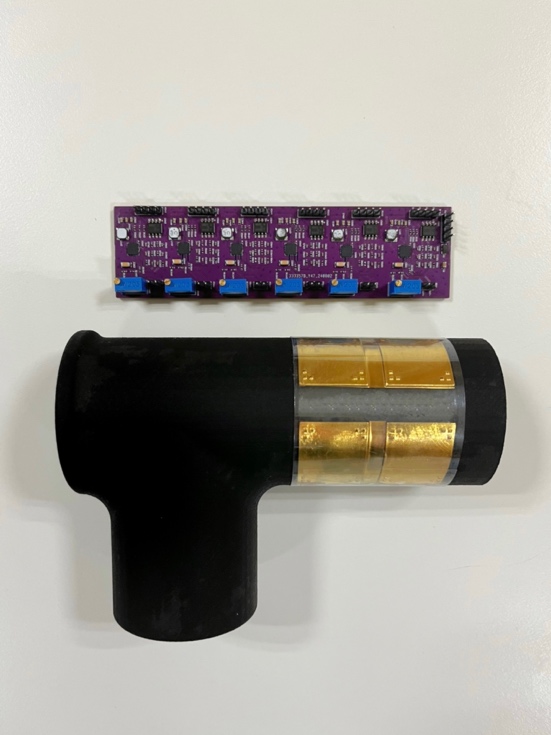

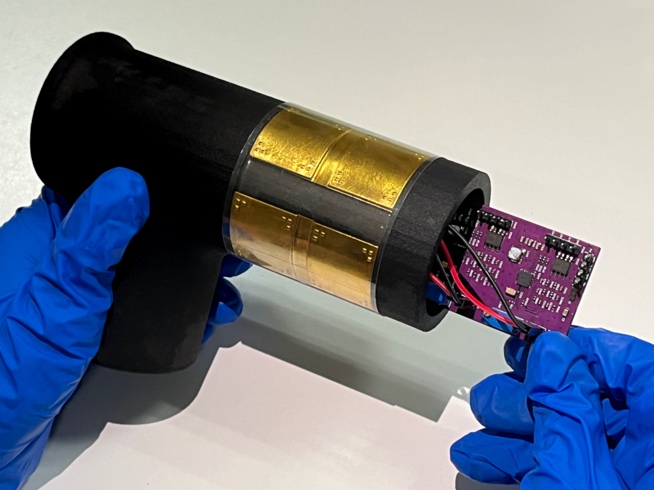
**

**Fig. S24.** Actuating circuitry for haptic interface in a cane.


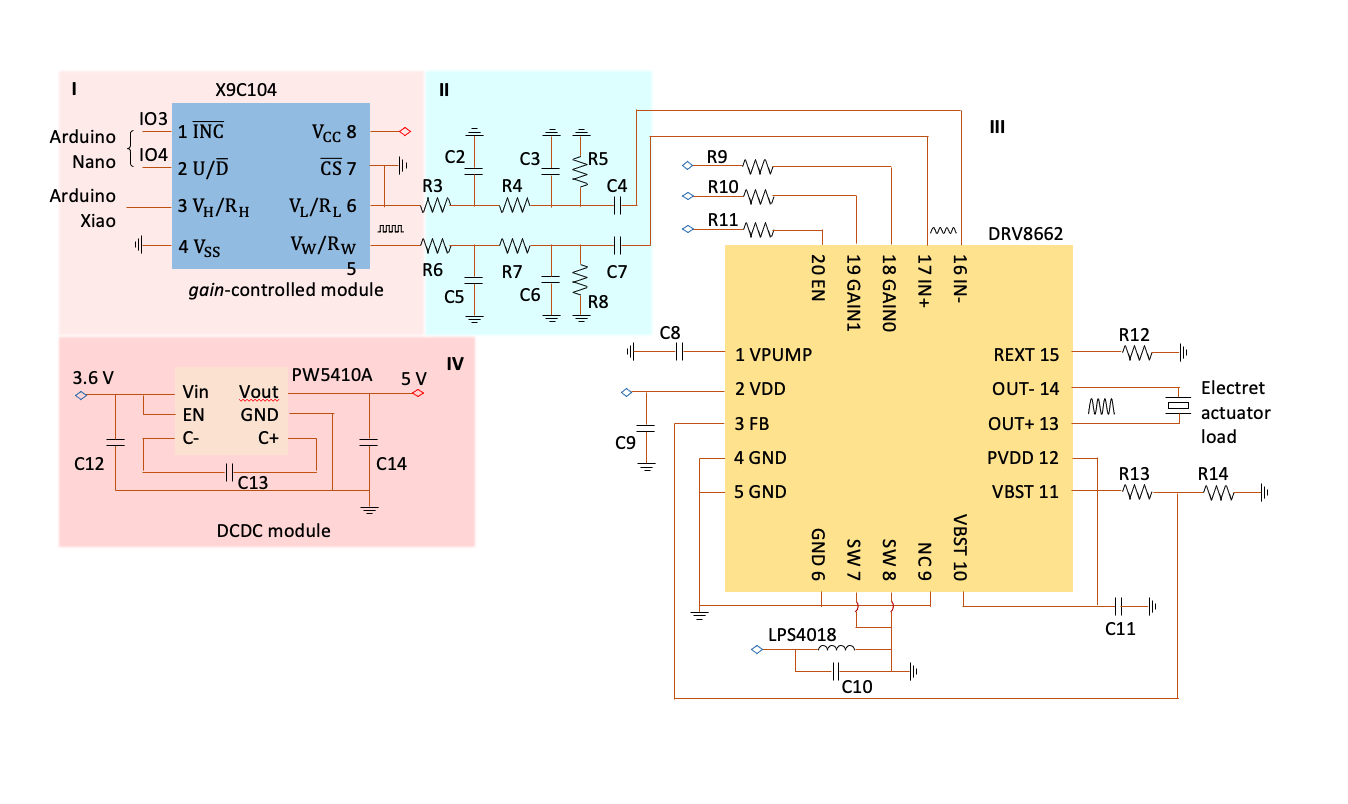


**Fig. S25.** Driving circuit schematic for haptic interface.


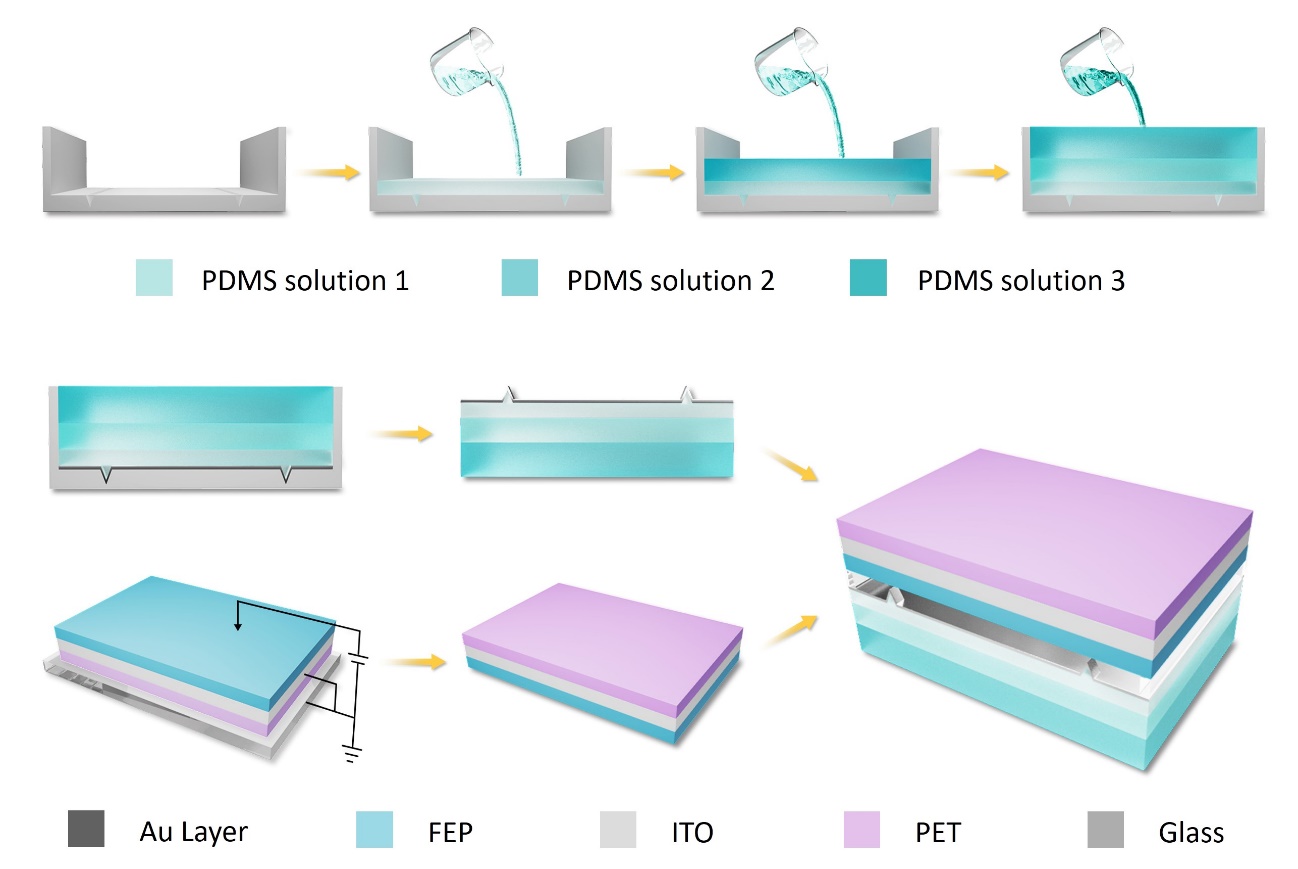


**Fig. S26.** Fabrication process of the electret actuator.


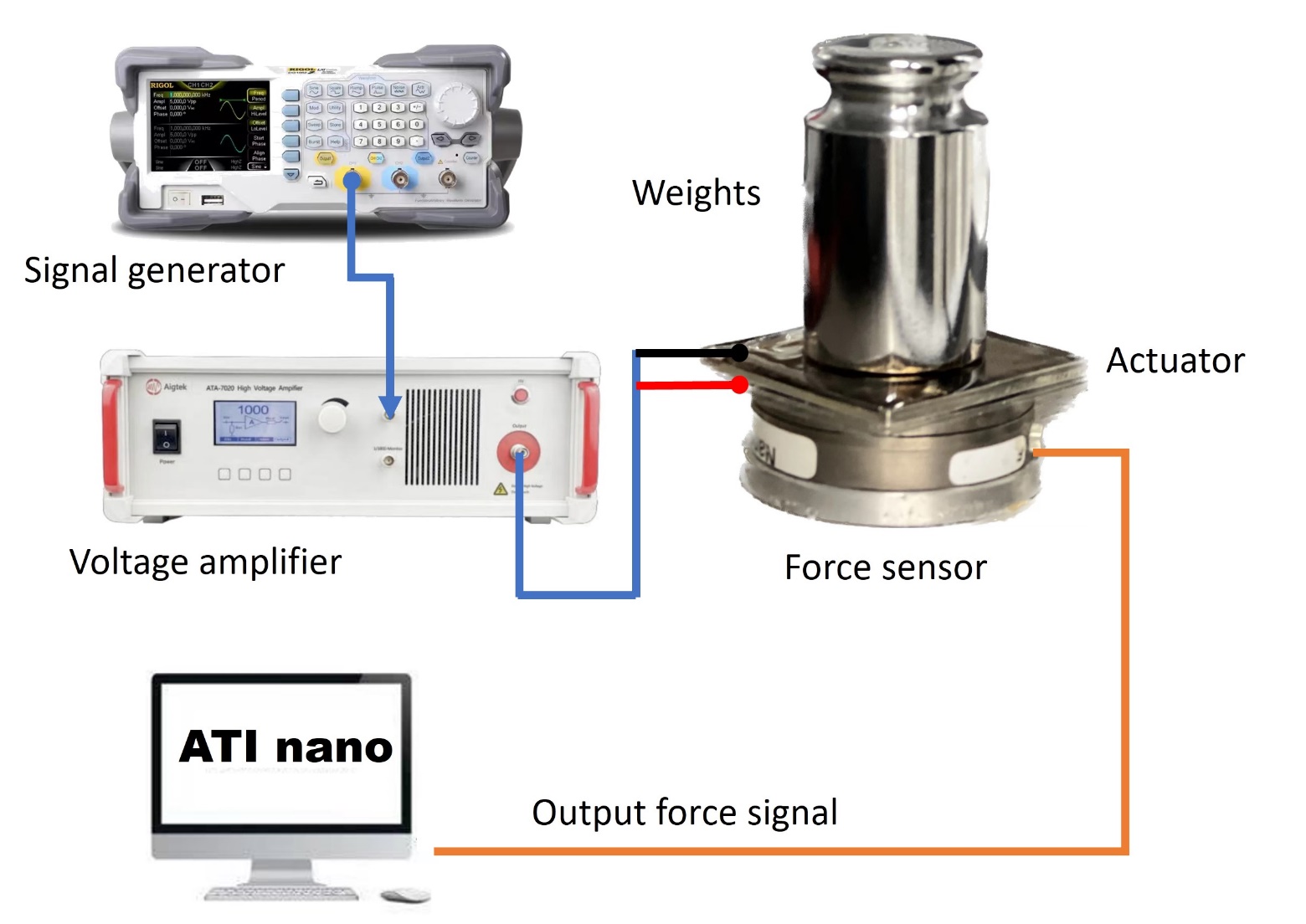


**Fig. S27.** Actuator output force characterization platform.
